# Human Subtelomeric WASH Genes Encode a New Subclass of the WASP Family

**DOI:** 10.1371/journal.pgen.0030237

**Published:** 2007-12-21

**Authors:** Elena V Linardopoulou, Sean S Parghi, Cynthia Friedman, Gregory E Osborn, Susan M Parkhurst, Barbara J Trask

**Affiliations:** 1 Division of Human Biology, Fred Hutchinson Cancer Research Center, Seattle, Washington, United States of America; 2 Division of Basic Sciences, Fred Hutchinson Cancer Research Center, Seattle, Washington, United States of America; Stanford University School of Medicine, United States of America

## Abstract

Subtelomeres are duplication-rich, structurally variable regions of the human genome situated just proximal of telomeres. We report here that the most terminally located human subtelomeric genes encode a previously unrecognized third subclass of the Wiskott-Aldrich Syndrome Protein family, whose known members reorganize the actin cytoskeleton in response to extracellular stimuli. This new subclass, which we call WASH, is evolutionarily conserved in species as diverged as *Entamoeba*. We demonstrate that WASH is essential in *Drosophila.* WASH is widely expressed in human tissues, and human WASH protein colocalizes with actin in filopodia and lamellipodia. The VCA domain of human WASH promotes actin polymerization by the Arp2/3 complex in vitro. WASH duplicated to multiple chromosomal ends during primate evolution, with highest copy number reached in humans, whose WASH repertoires vary. Thus, human subtelomeres are not genetic junkyards, and WASH's location in these dynamic regions could have advantageous as well as pathologic consequences.

## Author Summary

Human subtelomeres are rearrangement-prone regions near chromosome ends. They are concentrations of large, recent interchromosomal duplications. Over half of subtelomeric sequences changed copy number or location since humans and chimpanzee diverged, and subtelomeric content varies greatly among humans. Despite this dynamic activity, subtelomeres contain genes. We report the discovery of genes defining a previously unrecognized third subclass of the Wiskott-Aldrich Syndrome protein (WASP) family within human subtelomeres. The known WASP family members reorganize actin structures in cells in response to various signals, thereby causing cells to change shape and/or move. Representatives of this newly identified subclass, called WASH, exist in many other species, even in *Entamoeba* and slime mold. Like other WASP family members, WASH colocalizes with actin at the cell periphery and promotes actin polymerization in vitro. Flies lacking WASH die before becoming adults, demonstrating that WASH is critical for survival, and its function is distinct from that of the two other WASP subclasses, Wasp and Scar. Identification of the WASH subclass opens the way for future elucidation of WASH's role in the life cycles of diverse organisms, the implications of human variation in WASH copy number, and the consequences of WASH's location in dynamic telomere-adjacent regions.

## Introduction

Human chromosome termini are unusual in their sequence content and frequency of structural rearrangement. Their ends are capped with telomeres that protect chromosomes against degradation and fusion [1] and that can exert a silencing effect on nearby genes [2,3]. Patchworks of large DNA segments duplicated on various subsets of chromosomes lie just proximal of telomeres [4]. These subtelomeric regions comprise less than 0.1% of the human genome, but account for over 40% of interchromosomal duplicates in the genome assembly that formed since human and chimpanzee diverged. Other lines of evidence further support the notion that subtelomeres are hotspots of DNA breaks and repair [5–7].

While subtelomeric dynamics might disrupt gene function, they can also fuel rapid changes in subtelomeric gene repertoires. Subtelomeres of yeast and the malaria parasite, *Plasmodium*, harbor genes with important roles in adaptive processes [8,9]. Human subtelomeric genes vary in copy number and chromosomal distribution and include odorant and cytokine receptors, homeodomain proteins, secretoglobins, and other genes of unknown function [4]. Demonstrations of expression from more than one subtelomeric location for several human genes [4] suggest that interindividual variation in subtelomeric gene repertoires might underlie some phenotypic differences among humans. Indeed, subtle chromosomal rearrangements involving subtelomeres and neighboring chromosome-specific regions are detected in 3%–5% of individuals with unexplained mental retardation or developmental disorders [10]. However, no essential gene has yet been identified within human subtelomeres.

Here, we focus on the most telomerically duplicated human genes (currently arbitrarily named MGC52000), which heretofore had unknown function. A truncated copy of MGC52000 was first annotated in the pseudoautosomal region of Xqter/Yqter and called CXYorf1 [11]. A murine ortholog, orf19, encoding a 475-amino acid protein with similarity to proteins of unknown function from Drosophila melanogaster and Caenorhabditis elegans, was subsequently identified, but no motifs that would disclose its biological function were reported [12]. The C. elegans ortholog (Y48E1B.1) was recently named *ddl-2* for its involvement in life-span regulation and reported to have several proline-rich domains [13].

We now show that MGC52000 encodes a new member of the Wiskott-Aldrich Syndrome Protein (WASP) family and is conserved from *Entamoeba* to human. Known WASP family members participate in cytoskeleton reorganization and signal transduction by acting as effectors of Rho-GTPases and polymerizing actin via the Arp2/3 complex [14,15]. They are involved in cell motility, phagocytosis, and cytokinesis in diverse processes such as embryogenesis, angiogenesis, inflammatory immune response, microbial infection, and cancer metastasis [16–18]. Five WASP family members are currently known in mammals, and they fall into two subclasses, WASP/N-WASP and SCAR/WAVE [16,18]. We have renamed the human subtelomeric MGC52000 genes WASH, for Wiskott-Aldrich Syndrome Protein and SCAR Homolog.

Our comparative and phylogenetic analyses of the WASP family reveal that WASH proteins define a new subclass in the WASP family. We experimentally confirm that human WASH protein colocalizes with actin in cells and promotes Arp2/3-dependent actin polymerization in vitro and that WASH is transcribed in most tissues. We show that the single *Drosophila* WASH ortholog is essential for viability. It is therefore remarkable that human genomes harbor multiple functional WASH paralogs in highly dynamic subtelomeric regions.

## Materials and Methods

Details of materials and methods are provided in Text S1.

## Results

### Widespread Chromosomal Distribution and Expression of Human WASH Genes

**Figure 1:**
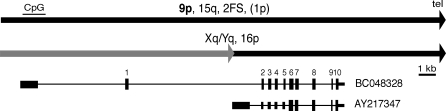
Organization of WASH Loci in the Current Genome Assembly and Representative Transcripts Only the 9p copy (bold) has a full-length intact ORF; others are partial or disrupted by frameshifts or in-frame stop codons. One copy assigned to 1p is more likely to be a variant allele of 19p [4]. Coding exons are numbered 1 through 10; thin bars represent non-coding exons. Sequence indicated in gray is shared only by Xq/Yq and 16p and lacks the N-terminal portion of WASH. Black and gray arrowheads indicate terminal and degenerate internal telomere-repeat arrays, respectively.

Seven copies of WASH are evident in the latest human genome assembly (March 2006). One is in 2q13–14 at the ancestral telomere–telomere fusion site (2qFS), and the others lie at different chromosomal ends (Figure 1). In contrast, the mouse genome contains only one ortholog (AJ304796) at an internal location on chromosome 17 (which we verified by FISH, not shown). One human cDNA in GenBank, BC048328, contains a full-length 468-aa open reading frame (ORF) with 86.3% amino-acid identity to the mouse ortholog. Human WASH genes have 11 exons and span 15 kbp, commencing in a 1-kb CpG island and ending within 5 kbp of the telomere array at four locations in the assembly (Figure 1). Only the 9p copy in the assembly encodes a full-length intact protein, while other copies are prematurely truncated by frame-shifts or in-frame stop codons. Three chromosome termini carry truncated copies lacking the first two exons, owing to past translocation events that transferred only the distal portion of the gene [4]. Spliced transcripts also lacking the proximal portion and containing a shorter ORF (264 aa) have been reported to GenBank (e.g., AY217347, Figure 1).

We detect spliced transcripts of human WASH by RT-PCR in all tissues tested using RT-PCR, Northern blot analysis, and a 72-tissue dot-blot (Figures S1–S3). According to publicly available array data [19], human WASH is expressed in a variety of tissues, with somewhat higher levels in blood cells and some areas of the brain.

### WASH Is an Evolutionarily Conserved New WASP Family Member

**Figure 2:**
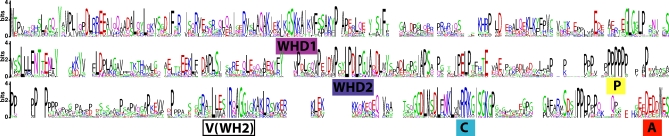
LogoPlot of Protein Alignment of WASH Orthologs from 21 Diverse Species from Mammals to *Entamoeba* Heights of letters indicate degree of conservation in units of maximum entropy expressed in bits. Colors indicate amino-acid similarity: KRH, green; DE, blue; and AVLIPWFM, red. Conserved domains discussed in the text are labeled at their approximate midpoints. Gaps indicate regions in the alignment where only a minority of the sequences has residues present. The actual alignment is provided in Figure S4.

**Figure 3:**
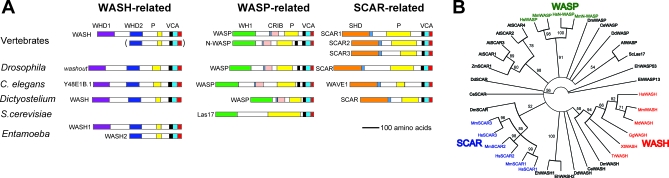
WASH Proteins Are Phylogenetically and Structurally Distinct from Known WASP Family Members (A) WASP family members and characteristic domains, adapted from reference [16] to show the new subfamily of WASH orthologs. Humans possess multiple WASH genes distributed in their subtelomeres, including short forms like one of two *Entamoeba* orthologs. Excluding *Entamoeba*, all other non-primate species examined have one WASH ortholog, except S. cerevisiae, in which there is only a WASP-related homolog. P, proline-rich domain; VCA, actin-binding and polymerizing domains; WHD1 and WHD2, subfamily-specific N-terminal domains of WASH (Figure S7). (B) Neighbor joining tree of WASP family members based on C-terminal (VCA region) alignment (Figure S6). Bootstrap values were calculated over 1,000 iterations. Vertebrate clades with high bootstrap support are in color. Species are indicated using two-letter abbreviations in front of each protein name (see Text S1).

In order to deduce the function of the WASH protein, we compared orthologous sequences that we identified from 21 diverse organisms (Figures 2, 3, and S4). The resulting multiple sequence alignments reveal high conservation of this protein among vertebrates. Regions of local high similarity throughout the protein, but especially in the C-terminal portion of the alignment, are apparent between predicted vertebrate WASH proteins and sequences of fly, worm, and even evolutionarily distant *Dictyostelium* and *Entamoeba*. Notably, one of the two *Entamoeba* WASH genes encodes a short protein, containing only the C-terminal portion, and is like the human short form, indicating its potential functional significance. Vertebrate WASH proteins have conserved predicted nuclear localization and export signals and sumoylation sites (Figure S5) suggesting a possible role in the nucleus.

The most highly conserved C-terminal regions of predicted WASH proteins show similarity to the actin-binding domain (WH2) of WASP family members (Figures 2, 3, and S4–S9). WASH proteins also have a proline-rich stretch (P), followed by the WH2 domain (V), a central region (C), and an acidic stretch (A) at the very C terminus. Together, these domains form the so-called VCA module found in all WASP family members [16] (Figure 3A). The VCA module is the minimal region required for binding and activation of actin polymerization via the Arp2/3 complex [14]. In known WASPs, the WH2 domain contains four conserved residues essential for actin binding [20], the A region has a conserved tryptophan residue and mediates binding to the Arp2/3 complex [14], and the amphipathic helical structure and conserved arginine in the C region induce conformational changes in Arp2/3 necessary to stimulate actin nucleation [21]. All these features are well conserved in WASH orthologs (Figures 2 and S4).

### WASH Proteins Define a New Subclass in the WASP Family

Mammalian WASH, WASP, and SCAR proteins separate into distinct clades supported by high bootstrap values in a phylogenetic tree (Figure 3B) based on the alignment of the VCA region of all known WASP family members (Figure S6). Based on their VCA sequences, WASP family orthologs from nonvertebrates do not cluster with statistical support into any one of these three clades. However, WASP family members from these organisms can be grouped together with the corresponding mammalian orthologs based on sequence homology within the N-terminal portion of the proteins (Figures 3A and S7).

Structural divergence of the known WASP family members outside the VCA region leads to variation in their activity and regulation and is used to subdivide them into WASPs and SCARs [15] (Figure 3A). WASP and N-WASP act as specific effectors of the small GTPase Cdc42 and have a WASP homology domain 1 (WH1) and a GTPase-binding domain (CRIB) at their N termini [22]. SCAR proteins lack these domains, but possess a specific SCAR-homology domain (SHD) and play a major role in Rac-induced actin dynamics [23].

WASH proteins lack N-terminal domains characteristic of either WASP or SCAR proteins, but they possess two evolutionarily conserved regions that appear to be specific to WASH orthologs (named WASH homology domains 1 and 2 (WHD1 and −2)) (Figures 2 and 3A). Based on the distinguishing features of the N-terminal portions and our phylogenetic analyses of VCA regions, we conclude that WASH proteins define a new WASP family subclass that is conserved from *Entamoeba* to human.

### Human WASH Colocalizes with Actin

**Figure 4:**
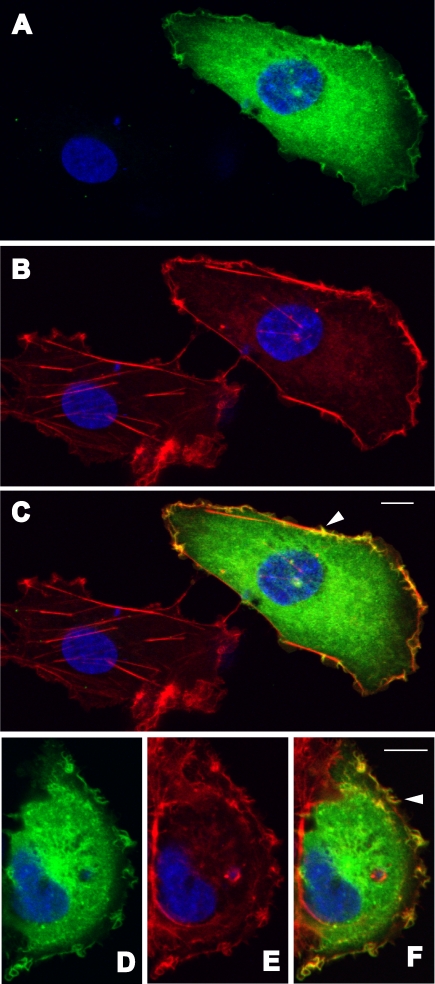
Colocalization of Transiently Expressed, GFP-Tagged Full-Length Human WASH with Actin in Filopodia (Two Indicated by Arrowheads) and Lamellipodia. (A) and (D), GFP-WASH (green) and DAPI to stain nuclei (blue); (B) and (E), Texas-red phalloidin to stain actin (red) and DAPI; (C) and (F), three-color overlay. Size bar, 10 μm.

Human WASH protein colocalizes with actin in vivo, consistent with our prediction that WASH has a role in actin polymerization and cytoskeleton reorganization (Figure 4). We transiently expressed full-length WASH corresponding to the BC048328 cDNA sequence and carrying GFP at its C terminus in COS-7 cells. This fusion protein colocalizes with actin in filopodia and lamellipodia, which are actin-rich cell surface extensions, but not in actin-rich stress fibers.

### WASH Functions As Actin Nucleation Promoting Factor

**Figure 5:**
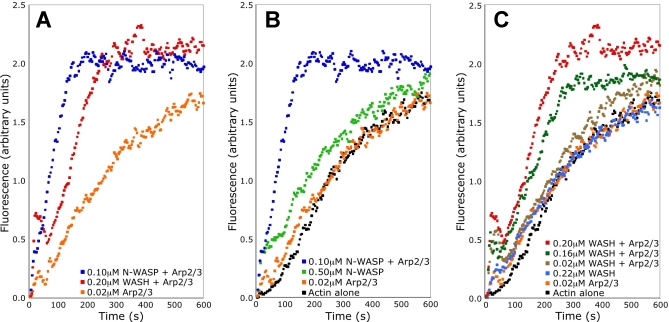
WASH Promotes Actin Nucleation by the Arp2/3 Complex Pyrene-actin polymerization assays were conducted with the concentrations indicated for WASH VCA and N-WASP VCA (A). Similar to N-WASP VCA (B), WASH VCA (C) does not promote actin nucleation in the absence of Arp2/3. In the presence of Arp2/3, WASH promotes actin nucleation in a concentration-dependent manner (C).

WASP family proteins are known to stimulate actin polymerization by the Arp2/3 complex. The VCA module of previously characterized WASP family members is necessary and sufficient for this activity [14]. In order to test the effect of WASH on actin polymerization by the Arp2/3 complex, we performed pyrene actin-polymerization assays using the VCA region of the human WASH and N-WASP proteins. Without the Arp2/3 complex, neither the WASH VCA nor the N-WASP VCA has an effect on actin polymerization (Figure 5B and 5C). The combination of WASH VCA and Arp2/3 complex strongly stimulates spontaneous assembly of monomeric actin (Figure 5A and 5C), leading to reduction of the initial lag associated with nuclei formation, and increasing the maximum rate of filament elongation, which is dependent on the concentration of growing ends. WASH's effect on actin polymerization in the presence of Arp2/3 is less robust than that of the N-WASP VCA (Figure 5A and 5B), but is similar to that of other family members like WASP and SCAR, which each induce unique kinetics of actin assembly [24]. We conclude that WASH, like the known WASP family members, is an endogenous activator of de novo actin filament assembly.

### *Drosophila* WASH Is Essential

The single WASH ortholog in *Drosophila*, which we have named *washout* (*wash*; CG13176), is structurally and phylogenetically distinct from the two WASP family members already characterized in this species (Figure 3A). Homozygous mutations in either Wasp or Scar result in zygotic lethality, although some of the Wasp mutants (“escapers”) survive until adulthood and appear morphologically normal, but are lethargic and passive in their behavior [25,26]. *wash* gene products, like those of Wasp and Scar, are provided maternally, and *wash* transcripts appear to be distributed uniformly throughout the early embryo, based on RNA in situ hybridizations performed by the Berkeley Drosophila Genome Project (BDGP) [27] (http://www.fruitfly.org/cgi-bin/ex/insitu.pl).

**Figure 6:**
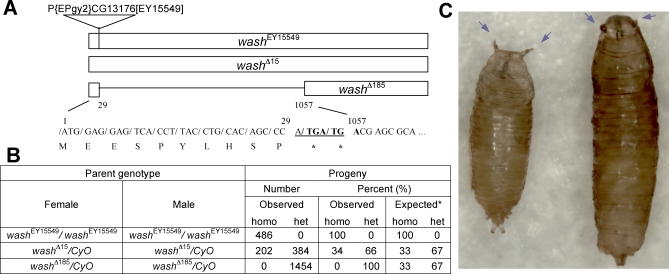
Analysis of *washout* Mutant Flies (A) A schematic of the *washout* coding region showing the original P-element insertion allele (*wash* ^EY15549^) and a precise (*wash* ^exc15^) and imprecise (*wash* ^Δ185^) excision allele. Sequence analysis of *wash* ^Δ185^ reveals two stop codons at positions 11 and 12 due to the internal deletion of 1,029 nucleotides and insertion of six nucleotides (underlined) at the junction. (B) Genetic analysis of *washout* alleles. No flies homozygous for the *wash* ^Δ185^ excision allele were recovered. *CyO* is a balancer chromosome that is homozygous lethal. (C) The *wash* ^Δ185^ mutant phenotype. Bright-field micrograph of pupae heterozygous (left) or homozygous (right) for the *wash* ^Δ185^ allele. The homozygous mutant displays an elongated phenotype and spiracle-eversion (arrows) defects, while the heterozygote is phenotypically normal.

To investigate *washout* function, we obtained a P-element insertion line, P{EPgy2}CG13176^EY15549^ (ref [28]; BDGP, unpublished data), with a P-element insertion at the beginning of the *washout* coding region. These flies are homozygous viable with no apparent phenotype. We generated numerous imprecise excision alleles of this insertion line that are lethal when homozygous. In one of the alleles, *wash* ^Δ185^, more than half of the coding region (up to the VCA module) is deleted and two stop codons at positions 11 and 12 are introduced (Figure 6A); no flies homozygous for this allele survive to adulthood (Figure 6B). Flies bearing precise excisions of the P-element insertion in the homozygous state were viable (Figure 6B), indicating that the recessive lethality of *wash* ^Δ185^ is due to disruption of *washout*.

To determine the stage at which lethality occurs in *wash* ^Δ185^ homozygous flies, we crossed flies carrying the *wash* ^Δ185^ allele to a balancer chromosome (a multiply inverted chromosome that suppresses recombination) carrying GFP, which allows for easy detection and separation of *wash* ^Δ185^ homozygous embryos. Analyses of 200 *wash* ^Δ185^ homozygous embryos revealed that the majority of them (98%) hatched, although none of them produced an adult fly. Further analysis showed that these *wash* ^Δ185^ animals die at the transition from 3rd larval instar to prepupal stage. While flies heterozygous for the *wash* ^Δ185^ allele have wild-type pupal morphology, *wash* ^Δ185^ homozygotes fail to contract their body and evert their spiracles prior to secreting their pupal cuticle, resulting in an elongated appearance (Figure 6C).

### WASH Genes Duplicated in Primate Genomes

**Figure 7:**
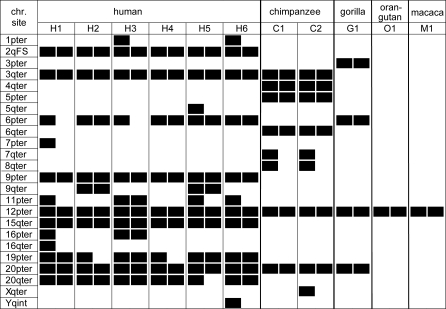
Summary of Locations of WASH Detected by FISH in Unrelated Individuals The signal-scoring criteria used to deem a location as homozygous (two bars) or heterozygous (one bar) for the presence of WASH sequence are supplied in Text S1. Here, we indicate human WASH locations excluding partial duplicates on Xqter, Yqter, and some 16pter alleles, which were detected only with FISH probes including the distal portion of WASH. For the nonhuman primates, we used a pool of probes completely spanning WASH in order to report all WASH locations in these species. Nonhuman primate chromosomes are numbered according to the corresponding chromosome in the human karyotype.

While a single-copy gene encodes WASH in most species analyzed, WASH genes experienced extensive duplication and dispersal to multiple chromosome ends during primate evolution. Our FISH assays detect many chromosomal sites, as well as extensive variation in the copy number and location of WASH genes between primate species and among human individuals (Figure 7). The total number of WASH copies, excluding partial duplications, ranges from >15 to >20 in the six analyzed human genomes. Collectively, these copies are found in 16 different sites and represent both WASH pseudogenes and intact WASH genes (see below). Our FISH analyses also support the conclusion that termini of 16p, Xq, and Yq typically carry a truncated WASH.

Nonhuman primates have far fewer copies of WASH than humans. WASH was detected at only eight or nine sites in the two chimpanzees analyzed by FISH and four sites in gorilla (Figure 7). WASH appears to be single-copy in orangutan and rhesus macaque, where it resides on 12p. Since the 12p location is shared among primates, it is likely to be the ancestral location of WASH before it duplicated during hominid diversification.

### Human Variation in WASH Coding Potential

Given the large number of human WASH loci detected by FISH and the relative paucity of sequenced WASH paralogs in public databases, we characterized the protein-coding potential of additional copies. We sequenced long-range PCR products encompassing coding exons 2–10 from three unrelated individuals. We have so far identified up to five potentially functional WASH variants and multiple pseudogenes per genome, although deeper sampling would be required to account for all copies detected by FISH in these individuals. Our sequence analyses also reveal that two of the eight human WASH copies captured in a panel of monochromosomal hybrid cell lines are full-length, intact ORFs.

Intact and null WASH alleles are segregating in the human population at some chromosomal loci. For example, the hybrid panel's chromosome 20 carries the two full-length ORFs, one mapping to each end by FISH, but the presence of WASH at 20qter is polymorphic (Figure 7). Furthermore, the chromosome-9 allele captured in a panel of monochromosomal hybrid cell lines contains a stop codon in coding exon 5, but is 99.9% identical to the sequenced 9pter allele in the genome assembly, which appears intact.

Our survey found a total of 12 different intact WASH ORFs, in addition to BC048328, assigned by our analyses to chromosome 20, and the 9p copy in the assembly. These 14 ORFs differ from each other across exons 2–10 by up to18 amino acids (≥95.8% identity) and one indel of three amino acids in the P region (Table S1). It is possible that some of these variants have slightly different functions, as they exhibit up to 11 nonconservative amino acid differences, although we detect no evidence for positive selection in mammalian WASH genes by PAML or K-estimator analyses (Text S1).

Intact, and possibly more divergent, copies of WASH might be found at other chromosomal ends in other individuals, since subtelomeres undergo interchromosomal sequence exchange [4]. Indeed, by evaluating the sequences of WASH copies in the current assembly, we detect two apparent exchange events that resulted in the transfer of different segments of several hundred base pairs from one chromosome to another (Figure S10). Thus, WASH genes evolve through a combination of acquired mutation, recombination between alleles and/or paralogs, and selection, and WASH repertoires can be expected to vary extensively among individuals.

## Discussion

This study shows that the most telomerically located human gene family encodes evolutionarily conserved proteins with orthologs in vertebrates, flies, worms, slime mold, and entamoeba. Human WASH colocalizes with actin filaments in lamellipodia and filopodia and stimulates actin polymerization in vitro, corroborating our sequence-based predictions that WASH orthologs are new members of the WASP family. Our sequence comparisons show that these newly identified WASH proteins form a previously unrecognized subclass in the WASP family, distinct from the known N-WASP/WASP and SCAR/WAVE proteins.

Known WASP family members have been implicated in the formation of lamellipodia and filopodia and in membrane-trafficking processes such as endocytosis, intracellular pathogen motility, and vesicle motility [16,18,29]. Yeast has only one WASP-related protein; its deletion causes a severe growth phenotype [30]. *WASp* and *SCAR* are important for cell-fate decisions and cell morphology, respectively, during *Drosophila* embryonic development [25,26]. Drosophila *WASp* is also essential for myoblast fusion [31–33]. We show here that the newly identified third WASP family member in *Drosophila*, *washout*, is essential, indicating that this gene also has an important role in early development and is not redundant with other WASP family members.

The *Drosophila* WASH protein was recently identified as a component of a nuclear complex containing various transcriptional factors and chromatin modifiers [34]. Consistent with this finding, the vertebrate WASH consensus sequence possesses predicted nuclear localization, nuclear export, and sumoylation signals (Figure S5). Actin and actin-binding proteins are found in the nucleus, where they might be involved in chromatin remodeling, RNA processing, or gene expression [15,35,36]. N-WASP has been shown to participate directly in transcriptional regulation [36,37] in addition to its role in the cytoplasm. It is therefore likely that WASH also has both nuclear and cytoplasmic functions.

We anticipate that loss of WASH gene function in humans will have pathological consequences, particularly since human WASH is ubiquitously expressed, with highest levels in hematopoietic tissues and some brain areas. Knockout of either N-WASP or Scar2, both of which are also ubiquitously expressed, is embryonic lethal in mice [38,39]. Scar1 is most highly expressed in brain, and, accordingly, Scar1-null mice exhibit defects in brain function [40]. The WASP gene defective in human Wiskott-Aldrich syndrome is expressed in hematopoietic cells [41]; its loss causes eczema, thrombocytopenia, and immunodeficiency [42]. Human WASH genes are at heightened risk for deletion and rearrangement, since subtelomeres are hotspots of meiotic interchromosomal sequence transfers [4]. Furthermore, somatic variation in subtelomeric organization and/or telomere length could influence WASH gene expression. WASH was reported to be overexpressed in a breast cancer cell line [43] and might, like overexpression of N-WASP and the SCARs, contribute to metastasis [17].

On the other hand, subtelomeric dynamics might contribute to normal human phenotypic variation and, more generally, to diversification of the WASP family. Subtle intra-species phenotypic variation might result from variation in WASH repertoires (gene number, location, and/or sequence). Intact and defective/missing WASH alleles segregate in the human population at some loci (e.g., 9pter, 20qter). We have shown that subtelomeric exchanges can create WASH loci that appear to combine sequence differences accrued by copies on different chromosomes. Finally, the identified human sequences with intact long ORFs differ from one another by as many as 11 nonconservative amino acid changes, raising the possibility that the encoded proteins might have slightly different functions.

Remarkably, the genes encoding this evolutionarily conserved protein multiplied within subtelomeric regions during primate evolution and thus might contribute to interspecies phenotypic differences. Maintenance of subtelomeric segmental duplications in yeast requires selective pressure [44]. Although genetic drift cannot be excluded as the explanation for the rapid recent expansion of WASH copies in the human lineage, WASH expansion might be important for fast evolving processes such as immune response and/or brain function.

The presence of WASH orthologs in *Entamoeba* also raises the possibility of WASH involvement in pathogenic infection. A number of unrelated pathogens hijack actin-polymerization pathways in host cells to facilitate their infection [45,46]. Rho GTPases and WASP family members are implicated in these pathogenic processes [45–47]. It is possible that the primate-specific subtelomeric expansion of WASH genes is associated with host response to pathogen infection.

Given the critical roles of the WASP family in diverse cellular processes, elucidation of the specific role(s) of this newly identified and evolutionarily conserved WASH subclass should provide important insights into actin dynamics in response to external signals. The location of WASH in highly dynamic human subtelomeric regions predisposes it to duplication, deletion, and rearrangement. Certain rearrangements could have pathological consequences if WASH plays as important a role in humans as it does in *Drosophila*, where it is essential. Further characterization of WASH protein function(s) should shed light on how WASH genes and their proximity to telomeres contribute to normal human variation as well as pathology.

## Supporting Information

Dataset S1 Sequences of WASH Gene Variants and the Computationally Derived, Predicted WASH Protein Sequences of Various Species in FASTA File Format (29 KB PDF)

Figure S1 RT-PCR Confirmation of Human WASH Expression Using exon 8 and exon 10 Primers L, liver; LN, lymph node; H, heart; FB, fetal brain; g, genomic DNA. Reverse transcriptase (RT)-negative controls were also performed for some tissues. The 0.3-kb band is the expected size for fully spliced WASH transcript.
(226 KB PDF)

Figure S2 Multiple Tissue Expression Array Autoradiograph after Hybridization Using a WASH Probe (A), the Corresponding Key to Tissues (B), and (C) Relative WASH Hybridization Signals Normalized to the Ubiquitin Signal, Organized by Tissue Type (743 KB PDF)

Figure S3 Northern Blot Showing Expression of Spliced WASH Transcripts in Multiple Human Tissues The ∼1.8-kb band corresponds to the expected size of fully spliced, full-length WASH transcripts. The same blot was probed with β-actin as a loading control (bottom). The intensities of the ∼1.8-kb WASH band in each tissue are expressed as a fraction of intensity of the β-actin band(s) in the numbers at the bottom of the gel. Where two numbers are given, the top one normalizes to the top actin band, and the bottom normalizes to the sum of the two actin bands.
(278 KB PDF)

Figure S4 Multiple Sequence Alignment of WASH Orthologs Conserved domains are named above the alignment, using same colors as in Figures 2 and 3A. Boxed residues in red letters are conserved in >50% of sequences, and the >50% consensus is indicated at the bottom. Amino acids showing conservation across >80% of the sequences are also shown at the bottom. Amino acids showing 100% conservation are noted against red background. In the consensus sequences, ! is I or V; $ is L or M; % is F or Y; and # is any of NDQEBZ. Asterisks mark conserved residues important for function in known WASP family members. The line “Change” gives the residues changed by nonsynonymous SNPs detected in our survey of human WASH paralogs; underlined changes are nonconservative.
(594 KB PDF)

Figure S5 Logo Plot of Vertebrate WASH Based on Alignment Provided in Figure S4
Positions of predicted sumoylation sites and nuclear localization and export signals (SUMO, NLS, NES) are indicated. Conserved domains are named above the alignment, using the same colors as used in Figure 3A; the domains extend beyond the labels. Heights of letters indicate degree of conservation in maximum entropy expressed in bits. Similar amino acids are given the same color: KRH, green; DE, blue; and AVLIPWFM, red. Gaps in the logo plots are regions with residues in only the minority of sequences.
(992 KB PDF)

Figure S6 Multiple Sequence Alignment of the VCA Region in the C-Terminal Portion of WASP Family Members See Figure S4 for details. Species are indicated using two-letter abbreviation before each protein name; the code is provided in Text S1 (note: Mm is Mus musculus, not Macaca mulatta).
(1.2 MB PDF)

Figure S7 Logo Plots of (A) WASH, (B) WASP/N-WASP, and (C) SCAR/WAVE Subclasses of the WASP Family to Illustrate Protein Conservation across Species and Differences among These Subclasses in Their N-Terminal Portions The logo plots are based on alignments provided in Figures S4, S8, and S9, respectively. Conserved domains are named above the alignment, using the same colors as used in Figure 3A; the domains extend beyond the labels. Heights of letters indicate degree of conservation in maximum entropy expressed in bits. Similar amino acids are given the same color: KRH, green; DE, blue; and AVLIPWFM, red. Gaps in the logo plots are regions with residues in only the minority of sequences.
(2.5 MB PDF)

Figure S8 Multiple Alignment of WASP and N-WASP Orthologs from Diverse Species See Figure S4 legend for details.
(402 KB PDF)

Figure S9 Multiple Alignment of SCAR (WAVE) Orthologs from Diverse Species See Figure S4 legend for details.
(485 KB PDF)

Figure S10 Percent Identity Plots for Pairs of WASH Genomic Sequences That Appear to Have Undergone Recent Sequence Exchange (Chromosomes 1 versus 9 and 9 versus 16, Blue and Green Lines, Respectively) The plot for chromosome 16 versus chromosome X (red lines) is shown for comparison. Percent identity is plotted in 500-bp windows moving in 10-bp increments. Exchange events appear as shifts to very high sequence similarity. These events are also detected by GeneConv (reference 7 in Text S1), with corresponding Bonferroni-corrected Karlin-Altschul *p*-values of 0.0016 (g1 setting) and 0.0065 (g0 setting). Position 0 is in intron 1 of WASH, 110 nt preceding the start of the 2nd coding exon, corresponding to the start of the partial duplication on 16 and X/Y. The end of the region shown is at nt position 4207 in intron 10.
(245 KB PDF)

Table S1 Locations of Sequence Variants Identified in 14 Different WASH ORFs That Appear to Be Intact, at Least in Coding Exons 2–10 We used the cDNA sequence BC048328 as reference; “-” indicates where a sequence is not different from BC048328. These variants were derived from chromosomes captured in a monochromosomal hybrid panel, certain chromosomes isolated by flow-sorting (20 or 9–12) from three individuals, and copies PCR amplified from genomic DNA (without chromosomal assignment) from the same individuals. Note: Two ORFs in one individual (with asterisks) were identical in coding sequence, but differed by 3% in intronic sequence implying that they derive from different chromosomal locations. n.a., not applicable; n.d., not determined. The SNP highlighted in blue is unconfirmed, as it was observed only once in this survey and is not a variant site in EST sequences in public databases. The variant sequences are provided in FASTA format in Dataset S1 and have been submitted to GenBank with accession numbers EU240546–EU240557.
(348 KB PDF)

Text S1 Supplementary Information and Methods (204 KB DOC)

### Accession Numbers

Genomic sequence variants of human WASH obtained in this study are available from the National Center for Biotechnology Information (NCBI) GenBank database (http://www.ncbi.nlm.nih.gov/sites/gquery) under accession numbers EU240546–EU240557.
