# Supplementary material for: Human Subtelomeric WASH Genes Encode a New Subclass of the WASP Family
Source: PLoS Genet. 2007 Dec 21;3(12):e237. doi: 10.1371/journal.pgen.0030237 (PMC2151093; doi:10.1371/journal.pgen.0030237)
Supplement: Supplementary file 1 [file pgen.0030237.sd001.pdf]

>Macaque WASH
MTPVRTQHSLAGQTYAVPLIQPDLRREEAVQQMADALQHLQKVSGDIFSR
ISQRVEQSRNQVQAIGEKVSLAQAKIEKIKGSKKAIKVFSSAKYPAPE
RLQEYGSIFTGAQDPGPQRRPRHRIQSKHRPLDERALQ
EKLKYFPVCVSAKLEPEDDAEEGLGGLPSNISSVSSLLLF
NTTENLYKKYVFLDPLAGAVTKTHVMLGAETEEKLFD
APLSISKREQLEQQVPENYFYVPDLGQVPEIDVPSYLPDLPGIAN
DLMYSADLGPGIAPSAPGTIPELPTFHTEVAEPLKSDLQDGVLT
APPPPPPPPPPPPAPEVLASAPPLPPSTA
APVGQGGRQDNSSSASPSSVQGAPKEVVDPSGGRATLLESI
RQAGGIGKAKLRSVKERKLEKKKQKEQEQVRA
TSQGGDLMSDLFNKLVMRRKGISGKGPGAGEGPGGAFARVSD
SIPPLPPPQQPQAEEDEDDWES

>Opossum WASH
MTQSLYLEGQAYSVPLIQPDLRREEAIQQVADALQYLQKVSGDIFSR
ISQRVETSRSQLQVIGERVSLAQAKIEKIKGSKKAIKVFSSAKYPAPD
RLQEYNSIFTGAQDPGFQRRIRHKVQSKHRPLDERALQ
EKLKYFPVCVSTRPQPEDEAEEGLGGLPSNINSLSSLLLF
NTTENLYKKYVFLDPLAGAVTKTHVTLGTETEKLFD
APLSISKREQLEQQVAENYFYVPDLGQVPEIDVPSYLPDLPGIAD
DLMYSADLGPGIAPSAPGTIPELPTFNTELAEPFKPELDDDVLV
APPPPPPPPPPPPPAPEVSVSAPPPPPLPQ
LTSAQPASVDSSSVSPSVHVPPKEVVDPSSGRATLLESI
RQAGGIGKAKLRSVKERKLEKKKQKEQEQVRA
TSQGGDLMSDLFNKLVMRRKGISGKGPGANTGEGPGGAFARMSD
SIPPLPMLQQPPGEEDEDEWES

>Fugu WASH
MTHKHYLEGQVYSVPLIQPDLRREEAVHQIADALLYLETISTDIFRR
QVSDSVEKNRHQLQRIGDRIRLAQARVDKIKGSKKATKVFSSAKYPAPD
RLQDYSSIFTEATDPSSHIRPRHRIRNKHRPFDEKALQ
DKLVYYPVCVSRKKKSEDETEEGLGSLPRNISSVSSLLLF
NTTENLYKKYVFLDPLAGAVTKTHTTLETEKEEKPFD
APLSIAKREQLERQTAESYFYVPDLGQVPEIDVPTYLPDLPGIAD
DLSYNADLGPGFAPSGPTHNIPELPSFTEESSLPGSQLQ
PNVPPPPPPPPPPEPVATAATAGALPPPPP
PPPPPPPPPVEVAQAPSSGPVSGAPTEVVQPSDGRASLLESI
RNAGVIGKAKLRNMKERKMEKKKQKEQEQAVGV
ASSGGDFMSDLFNKLAMRRKGISGKGPAGSEASDAPASTGGAFARMSD
VIPPLPAPHSTTDDDDWEA

>Anopheles WASH
MHTYSVPIVKHDIRHEETILDAINAFEYLNQVVEDVFGK
INARIERNRARLEAIDGRVEQVSASVRKLHETKQAIIIYSPSRYPAAD

VDPTIEPTFTPTNGIALAECHFRLRDKPYLPSHSLQ
EKIQFYHVKSPRESERIFPFESAPARSGSGSLQGRGLGPFLDTLLLF
SEPAYVQQYRGQDWKRPVRKSSSHAVGGIREEKVLS
SSSPIIRSRMKKKSQDIFYTPSLNDAPKLDVPIDLPDLPGIVS
NINYVGNSTLIAPSLQLAGIIEQLPSLEDLTSAIDVADHHSK
EKKVGETPPPPPPPPPPPPPP
QPVVETATAPATSKPEAPKQKEPLPAPAGNAHFNLMEAI
RRAGGAEKANLRHSDRREGGGGRDGAAS
SGEPKSLIDDLHNKLQMRRKGISGTREPQEPQGNVIDRLSA
LIPPPAPKLAADASASESNEEDWE

>D.yakuba WASH
MEESPYLHSPYQVAIIATDLHHEDTIIQAAQSLDCLHKTINSIFER
IDARLARNGSKVEDINNRVKRAQAKIDALVGSKRAIQIFAPARFPASD
VLAPLPATFPQVAANPLMEQQVDQQPQRPYSSHSPAEQQQDD
ADIFFHVRDDREQESPLVAERKVTNRTAGLGSLPVGGVRSVPSLMRF
NTNEFAYGEDLNAWKRSLPPQNARRVASQSARLTAEKQLAP
APHSLAHGTTKLATPAGDLRYNPAAMAAPAIDVPLDLPDLPGIAN
DLQYEPVEEKTPIAPSQQFGDLPELPDLGLEEQEITVQAIAARTHIP
GPARKKSLGQCPSPVMAAPPPPPPPPPPPPPPPPA
QTMGIPSPPPFPTKGAVKPLSPSISTPLNMPPPPPATEDPRSELMAAI
RNAGGVHGGRLRSPAAAPLDVVDNTRSKAG
AAATGDLMADLHNKLMLRRKGISGSQNPGEATAGNPLMQQLSR
VIPPPVQPRKGSQSSDEAHSEDDEDGWN

>D.simulans WASH
MEESPYLHSPYQVAIIATDLHHEDTIIQAAQSLDCLHKTINSIFER
IDARLARNGSKVEDINNRVKRAQAKIDALVGSKRAIQIFAPARFPASD
VLAPLPATFPQVAANPLMEQPVEQKPQGSYSSHSPAEHKPDD
ADIFFHVRGDREQESPLVAERKITNRTAGLGSLPAGGVRSVPSLMRF
NTNEFAYGEDLNAWKRSLPPQSARRVASQSTQLTVDKQLAP
APHSLAHGTTKLATPAGDLRYNPAALAAPAIDVPLDLPDLPGIAN
DLQYEPVEEQTPIAPSQQFGDLPELPDLGLEEQVITVQAIAAQTHIP
GPLRRKSVGQCPSPVTAAPPPPPPPPPPPPPPPA
QTNAIPSPPPFPTKGAVKPLSPSLATPLNMPQPPPATEDPRSELMAAI
RNAGGVHGGRLRSPAAAPLDVVDNSRSKAG
GAATGDLMADLHNKLMLRRKGISGSQNPAEATAGNPLMQQLSR
VIPPPVQPRKGSQSSGEEHSEDDEDGWN

>CfNWASP
MSSGQQQPPPPRRVTNVGSLLLTPQENESLFTFLGKKCV
TMSSAVVQLYAADRNCMWSKKCSGVACLVKDNPQRSYFLRIFDIKDG
KLLWEQELYNNFVYNSPRGYFHTFAGDTCQVALNFANEEEAK
KFRKAVTDLLGRRQRKSEKRRDPPNGPNLP
MATVDIKNPEITTNRFYGPQVNNISHTKEKKKG
KAKKKRLTKADIGTPSNFQHIGHVGWDPNTGFDLNNLDPEL

KNLFDLCGISEAQLKDRETSKVIYDFIEKTGGVEAVKNELRRQA
PPPPPPSRGGPPPPPPPPHSSGPPPPPARGRGAPP
PPPSRAPTAAPPPPPPSRPGVGVPPPPPNRMYPPPPPALPSSA
PSGPPPPPPPLSLAGSAAPPPPPPPPPPPGPPPPPG
LPSDGDHQVPTPAGSKAALLDQIREGAQLKKVEQNSRPVSCSG
RDALLDQIRQGIQLKSVSDGQESTPPTPAPTSGIVGALMEVMQKR
SKAIHSSDEDEDEDDEEDFEDDDEWED

>XtSCAR2
MPLVARCIEPRFLCRQKLPPVRSELECMTNLTLSNVIRQLGSLSR
YAEDIFGELFSEASMFAHRVGSLGERVERLQVKVTQLDPKEEEVSLQGIN
TRKAFRSNTSQDQQLFIRDSLSLPVRETYSTCDMPPPLNILSPYREDE
KEALKFYTDPSYFFDLWKEKMLQDTKDIIKEKRKHRREKKDNPN
RGNVNPRKIRTRKEEWEKMKMGLEFVEAKDKMQNAGKF
FLLIGCSRLLLLGKVCTFYYTTRYMF
KHCFTKKNNFKCFFDSFSGQPSLHRSS
LVSPAYPPPAPPIGSPIGSRPSFSPPPAPPPPPPDG
SVPDAPYLPPPSPPPVFPSLPG
FPAPAPPPPPPAPTHLDYPAASPHSPTQAPSGG
PPPPPPPPPPPPGPPPPPAFSHSEGES
PSQISKPKTSLPPVSDPRSDLLSAIRE
GFQLRKVEEKMEHEKRDVGGNDVATILSRRIAVEYSDSEDDSSEF
DEDDWSD

>XtSCAR3
MPLVKRNIEPRHLCRGTVPDGVTSELECVTNSTLAAIIKQLGSLSR
HAEDIFGELFNEANSFYMRMNSLQERVDLLVIKVTQLDSTVEEVSLQDIN
MRKAFKSSTVQNQQVVSRNSIPNPVMEMYQRGDKPPPLNILTPYRDDK
KDGLRFYTDPSYFFNLWKEKMLQATEDKRKEKRRQKEQRLVEDPT
REVKKVRKARNRRLEWNMMAYDKELRPDNRFSPSPYHVASS
EGSLSPDNRQVPHIMYLDHSYPASPNHPG
QQIMAPAALLTSENKDIILMATQAQEHGYRSSAPGSRQNS
LNRAQQAHAPQQPEAIPNGPRPQVMKDYGGQ
QVPMTEYFVPPAPPPPPPVIPSAQTA
FDSPISAPTPVAPNIAALSSYIPAPPPAPPCPYSGSPPQT
GPVGPPVAPPPPPPGPPIITASPAHSAS
PPTLTVEPRKPQIPIIPMSDARSDLLAAIRR
GIQLRKVQEQREQEAKKEPVGNDVATILSRRIAVEYSESDDDSEL
EENEWSD

>GM10494.fs9-12.1
gagcagagccggagccaggtgcaggccattggagagaaggtctccttggcccaggccaag
attgagaagatcaagggcagcaagaaggccatcaaggtgttctccagtgccaagtaccct
gctccagagcgcctgcaggaatatggctccatcttcaccggcgcccaggaccctggcctg
cagagacgcccccgccacaggatccagagcaagcaccgcccccctggacgagcgggccctg
caggagaagctgaaggactttcctgtgtgcgtgagcaccaagccggagcccgaggacgat

gcagaagagggacttgggggtcttcccagcaacatcagctctgtcagctccttgctgctc
ttcaacaccaccgagaacctgtacaagaagtatgtcttcctggacccccctggctggtgct
gtaacaaagacccatgtgatgctgggggcagagacagaggagaagctgtttgatgccccc
ttgtccatcagcaagagagagcagctggaacagcaggtcccagagaactacttctatgtg
ccagacctgggccaggtgcctgagattgatgttccatcctacctgcctgacctgtccggc
attgccaatgacctcatgtacattgccgacctgggccccggcattgcccccctctgcccct
ggcaccattccagaactgcccaccttccacactgaggtagccgagcctctcaaggtagac
ctacaagatggggtactaaca---------ccacccccaccgcccccaccaccaccccca
gctcctgaggtgctggccagtgcacccccactcccaccctcaaccgcggcccctgtaggc
caaggcgccaggcaggacgacagcagcagcagcgcgtctccttcagtccagggagctccc
agggaagtggtcgacccctccggtggctgggcctctctgctagagtccatccgccaagct
gggggcatcggcaaggccaagctgcgcagcatgaaggagcgaaaggtggagaagaagaag
cagaaggagcaggagcaagtgagagccacgagccaaggtgggcacttgatgtcggatctc
ttcaacaagctggtcatgaggcgcaagggcatctctgggaaaagacctggggctggtgag
gggcccggaggagcctttgcccgtgtgtcagactccatccctcctctgccgccaccgcag
cagccacaggcagaggaggacgaggacgac

>GM10540.fs9-12.1
gagcagagccggagccaggtgctggccattggagagaaggtctccttggcccaggccaag
attgagaagatcaagggcagcaagaaggccatcaaggtgttctccagtgccaagtaccct
gctccagagcgcctgcaggaatatggctccatcttcaccggcgcccaggaccctggcctg
cagagacgcccccgccacaggatccagagcaagcaccgcccccctggacgagcgggccctg
caggagaagctgaaggactttcctgtgtgcgtgagcaccaagccggagcccgaggacgat
gcagaagagggacttgggggtcttcccagcaacatcagctctgtcagctccttgctgctc
ttcaacaccaccgagaacctgtacaagaagtatgtcttcctggacccccctggctggtgct
gtaacaaagacccatgtgatgctgggggcagagacagaggagaagctgtttgatgccccc
ttgtccatcagcaagagagagcagctggaacagcaggtcccagagaactacttctatgtg
ccagacctgggccaggtgcctgagattgatgttccatcctacctgcctgacctgtccggc
attgccaatgacctcatgtacattgccgacctgggccccggcattgcccccctctgcccct
ggcaccattccagaactgcccaccttccacactgaggtagccgagcctctcaaggtagac
ctacaagatggggtactaaca---------ccacccccaccgcccccaccaccaccccca
gctcctgaggtgctggccagtgcatccccactcccaccctcaaccgcggcccctgtaggc
caaggcgccaggcaggacgacagcagcaccagcgcgtctccttcagtccagggagctccc
agggaagtggtcgacccctccggtggctgggcctctctgctagagtccatccgccaagct
gggggcatcggcaaggccaagctgcgcagcatgaaggagcgaaaggtggagaagaagaag
cagaaggagcaggagcaagtgagagccacgagccaaggtgggcacttgatgtcggatctc
ttcaacaagctggtcatgaggcgcaagggcatctctgggaaaagacctggggctggtgag
gggcccggaggagcctttgcccgcgtgtcagactccatccctcctgtgccgccaccgcag
cagccacaggcagaggaggacgaggacgac

>GM07791.fs9-12.1
gagcagagccggagccaggtgcaggccattggagagaaggtctccttggcccaggccaag
attgagaagatcaagggcagcaagaaggccatcaaggtgttctccagtgccaagtaccct
gctccagagcgcctgcaggaatatggctccatcttcaccggcgcccaggaccctggcctg
cagagacgcccccgccacaggatccagagcaagcaccgcccccctggacgagcgggccctg
caggagaagctgaaggactttcctgtgtgcgtgagcaccaagccggagcccgaggacgat

gcagaagaggggcttgggggtcttcccagcaacatcagctctgtcagctccttgctgctc
ttcaacaccaccgagaacctgtacaagaagtatgtcttcctggacccccctggctggtgct
gtaacaaagacccatgtgatgctgggggcagagacagaggagaagctgtttgatgccccc
ttgtccatcagcaagagagagcagctggaacagcaggtcccagagaactacttctatgtg
ccagacctgggccaggtgcctgagattgatgttccatcttacctgcctgacctgtccggc
attgccaatgacctcatgtgcattgccgacctgggccccggcattgcccccctctgcccct
ggcaccattccagaactgcccaccttccacactgaggtagccgagcctctcaaggtagac
ctacaagatggggtactaaca---------gcaccaccaccgcccccaccaccacccccca
gctcctgaggtgctggccagtgcatccccactcccaccctcaaccgcggcccctgtaggc
caaggcgccaggcaggacgacagcagcagcagcgcgtctccttcagtccagggagctccc
agggaagtggtcgacccctccggtggctgggccactctgctagagtccatccgccaagct
gggggcatcggcaaggccaagctgcgcagcatgaaggagcgaaaggtggagaagaagaag
cagaaggagcaggagcaagtgagagccacgagccaaggtgggcacttgatgtcggatctc
ttcaacaagctggtcatgaggcgcaagggcatctctgggaaaagacctggggctggtgag
gggcccggaggagcctttgcccgcgtgtcagactccatccctcctctgccgccaccgcag
cagccacaggcagaggaggacgaggacgac

>GM10494.genomic.1
gagcagagccggagccaggtgcaggccattggagagaaggtctccttggcccaggccaag
attgagaagatcaagggcagcaagaaggccatcaaggtgttctccagtgccaagtaccct
gctccagagcgcctgcaggaatatggctccatcttcaccggcgcccaggaccctggcctg
cagagacgctcccgccacaggatccagagcaagcaccgccccctggacgagcgggccctg
caggagaagctgaaggactttcctgtgtgcgtgagcaccaagccggagcccgaggacgat
gcagaagagggacttgggggtcttcccagcaacatcagctctgtcagctccttgctgctc
ttcaacaccaccgagaacctgtacaagaagtatgtcttcctggacccccctggctggtgct
gtaacaaagacccatgtgatgctgggggcagagacagaggagaagctgtttgatgccccc
ttgtccatcagcaagagagagcagctggaacagcaggtcccagagaactacttctatgtg
ccagacctgggccaggtgcctgagattcatgttccatcctacctgcctgacctgcccggc
attgccaatgacctcatgtacattgccgacctgggccccggcattgcccccctctgcccct
ggcaccattccagaactgcccaccttccacactgaggtagccgagcctctcaaggtagac
ctacaagatggggtactaaca---------ccacccccaccgcccccaccaccacccccca
gctcctgaggtgctggccagtgcacccccactcccaccctcaaccgcggcccctgtaggc
caaggcgccaggcaggacgacagcagcagcagcgcgtctccttcagtccagggagctccc
agggaagtggtcgacccctccggtggctgggccactctgctagagtccatccgccaagct
gggggcatcggcaaggccaagctgcgcagcatgaaggagcgaaaggtggagaagaagaag
cagaaggagcaggagcaagtgagagccacgagccaaggtgggcacttgatgtcggatctc
ttcaacaagctggtcatgaggcgcaagggcatctctgggaaaagacctggggctggtgag
gggcccggaggagcctttgcccgcgtgtcagactccatccctcctgtgccgccaccgcag
cagccacaggcagaggaggacgaggacgac

>GM10540.fs20.1_and_GM10540.genomic.2
gagcagagccggagccaggtgcaggccattggagagaaggtctccttggcccaggccaag
attgagaagatcaagggcagcaagaaggccatcaaggtgttctccagtgccaagtaccct
gctccagagcgcctgcaggaatatggctccatcttcaccagcgcccaggaccctggcctg
cagagacgcccccgccacaggatccagagcaagcaccgccccctggacgagcgggccctg
caggagaagctgaaggactttcctgtgtgcgtgagcaccaagccggagcccgaggacgat

gcagaagagggacttgggggtcttcccagcaacatcagctctgtcagctccttgctgctc
ttcaacaccaccgagaacctgtacaagaagtatgtcttcctggacccccctggctggtgct
gtaacaaagacccatgtgatgctgggggcagagacagaggagaagctgtttgatgccccc
ttgtccatcagcaagagagagcagctggaacagcaggtcccagagaactacttctatgtg
ccagacctgggccaggtgcctgagattgatgttccatcctacctgcctgacctgtctggc
attgccaatgacctcatgtacattgccgacctgggccccggcattgcccccctctgcccct
ggcaccattccagaactgcccaccttccacactgaggtagccgagcctctcaaggtagac
ctacaagatggggtactaaca---------ccacccccaccgcccccaccaccaccccca
gctcctgaggtgctggccagtgcacccccactcccaccctcaaccgcggcccctgtaggc
caaggcgccaggcaggacgacagcagcagcagcgcgtctccttcagtccagggagctccc
agggaagtggtcgacccctccggtggctgggccactctgctagagtccatccgccaagct
gggggcatcagcaaggccaagccgcgcagcatgaaggagcgaaagctggagaagaagcag
cagaaggagcaggagcaagtgagagccacgagccaaggtgggcacttgatgtcggatctc
ttcaacaagctggtcatgaggcgcaagggcatctctgggaaaggacctggggctggtgag
gggcccggaggagcctttgcccgcgtgtcagactccatccctcctctgccgccaccgcag
cagccacaggcagaggaggacgaggacgac

>GM10540.genomic.1
gagcagagccggagccaggtgcaggccattggagagaaggtctccttggcccaggccaag
attgagaagatcaagggcagcaagaaggccatcaaggtgttctccagtgccaagtaccct
gctccagagcgcctgcaggaatatggctccatcttcaccagcgcccaggaccctggcctg
cagagacgcccccgccacaggatccagagcaagcaccgcccccctggacgagcgggccctg
caggagaagctgaagtactttcctgtgtgcgtgagcaccaagccggagcccgaggacgat
gcagaagagggacttgggggtcttcccagcaacatcagctctgtcagctccttgctgctc
ttcaacaccaccgagaacctgtacaagaagtatgtcttcctggacccccctggctggtgct
gtaacaaagacccatgtgatgctgggggcagagacagaggagaagctgtttgatgccccc
ttgtccatcagcaagagagagcagctggaacagcaggtcccagagaactacttctatgtg
ccagacctgggccaggtgcctgagattgatgttccatcctacctgcctgacctgtctggc
attgccaatgacctcatgtacattgccgacctgggccccggcattgcccccctctgcccct
ggcaccattccagaactgcccaccttccacactgaggtagccgagcctctcaaggtagac
ctacaagatggggtactaaca---------ccacccccaccgcccccaccaccaccccca
gctcctgaggtgctggccagtgcacccccactcccaccctcaaccgcggcccctgtaggc
caaggcgccaggcaggacgacagcagcagcagcgcgtctccttcagtccagggagctccc
agggaagtggtcgacccctccggtggctgggccactctgctagagtccatccgccaagct
gggggcatcagcaaggccaagccgcgcagcatgaaggagcgaaagctggagaagaagcag
cagaaggagcaggagcaagtgagagccacgagccaaggtgggcacttgatgtcggatctc
ttcaacaagctggtcatgaggcgcaagggcatctctgggaaaggacctggggctggtgag
gggcccggaggagcctttgcccgcgtgtcagactccatccctcctctgccgccaccgcag
cagccacaggcagaggaggacgaggacgac

>chr9 assembly
gagcagagccggagccaggtgcaggccattggagagaaggtctccttggcccaggccaag
attgagaagatcaagggcagcaagaaggccatcaaggtgttctccagtgccaagtaccct
gctccagggcgcctgcaggaatatggctccatcttcacgggcgcccaggaccctggcctg
cagagacgcccccgccacaggatccagagcaagcaccgcccccctggacgagcgggccctg
caggagaagctgaaggactttcctgtgtgcgtgagcaccaagccggagcccgaggacgat

gcagaagagggacttgggggtcttcccagcaacatcagctctgtcagctccttgctgctc
ttcaacaccaccgagaacctgtacaagaagtatgtcttcctggacccccctggctggtgct
gtaacaaagacccatgtgatgctgggggcagagacagaggagaagctgtttgatgccccc
ttgtccatcagcaagagagagcagctggaacagcaggtcccagagaactacttctatgtg
ccagacctgggccaggtgcctgagatccatgttccatcctacctgcctgacctgcccggc
attgccaacgacctcatgtacagtgccgacctgggccccggcattgcccccctctgcccct
ggcaccattccagaactgcccaccttccacactgaggtagccgagcctctcaaggtagac
ctacaagatggggtactaaca---------ccacccccaccgcccccaccaccaccccca
gctcctgaggtgctggccagtgcacccccactcccaccctcaaccgcggcccctgtaggc
caaggcgccaggcaggacgacagcagcagcagcgcatctccttcagtccagggagctccc
agggaagtggtcgacccctccggtggctgggccactctgctagagtccatccgccaagct
gggggcatcggcaaggccaagctgcgcagcatgaaggagcgaaagctggagaagcagcag
cagaaggagcaggagcaagtgagagccacgagccaaggtgggcacttgatgtcggatctc
ttcaacaagctggtcatgaggcgcaagggcatctctgggaaaggacctggggctggtgag
gggcccggaggagcctttgtccgcgtgtcagactccatccctcctttgccgccaccgcag
cagccacaggcagaggaggacgaggacgac

>GM10494.genomic.3
gagcagagccggagccaggtgcaggccattggagagaaggtctccttggcccaggccaag
attgagaagatcaagggcagcaagaaggccatcaaggtgttctccagtgccaagtaccct
gctccagagcgcctgcaggaatatggctccatcttcaccgAcgcccaggaccctggcctg
cagagacgcccccgccacaggatccagagcaagcaccgccccctggacgagcgggccctg
caggagaagctgaaggactttcctgtgtgcgtgagcaccaagccggagcccgaggacgat
gcagaagagggacttgggggtcttcccagcaacatcagctctgtcagctccttgctgctc
ttcaacaccaccgagaacctgtacaagaagtatgtcttcctggacccccctggctggtgct
gtaacaaagacccatgtgatgctgggggcagagacagaggagaagctgtttgatgccccc
ttgtccatcagcaagagagagcagctggaacagcaggtcccagagaactacttctatgtg
ccagacctgggccaggtgcctgagattgatgttccatcctacctgcctgacctgcccggc
attgccaacgacctcatgtacattgccgacctgggccccggcattgcccccctctgcccct
ggcaccattccagaactgcccaccttccacactgaggtagccgagcctctcaaggtagac
ctacaagatggggtactaaca---------ccacccccaccgcccccaccaccaccccca
gctcctgaggtgctggccagtgcacccccactcccaccctcaaccgcggcccctgtaggc
caaggcgccaggcaggacgacagcagcagcagcgcatctccttcagtccagggagctccc
agggaagtggtcgacccctccggtggccgggccactctgctagagtccatccgccaagct
gggggcatcggcaaggccaagctgcgcagcatgaaggagcgaaaggtggagaagcagcag
cagaaggagcaggagcaagtgagagccacgagccaaggtgggcacttgatgtcggatctc
ttcaacaagctggtcatgaggcgcaagggcatctctgggaaaggacctggggctggtgag
gggcccggaggagcctttgcccgcgtgtcagactccatccctcctctgccgccaccgcag
cagccacaggcagaggaggacgaggacgac

>GM10494.genomic.2
gagcagagccggagccaggtgcaggccattggagagaaggtctccttggcccaggccaag
attgagaagatcaagggcagcaagaaggccatcaaggtgttctccagtgccaagtaccct
gctccagagcgcctgcaggaatatggctccatcttcacgggcgcccaggaccctggcctg
cagagacgcccccgccacaggatccagagcaagcaccgccccctggacgagcgggccctg
caggagaagctgaaggactttcctgtgtgcgtgagcaccaagccggagcccgaggacgat

```
gcagaagagggacttgggggtcttcccagcaacatcagctctgtcagctccttgctgctc
ttcaacaccaccgagaacctgtacaagaagtatgtcttcctggacccccctggctggtgct
gtaacaaagacccatgtgatgctgggggcagagacagaggagaagctgtttgatgccccc
ttgtccatcagcaagagagagcagctggaacagcaggtcccagagaactacttctatgtg
ccagacctgggccaggtgcctgagattcatgttccatcctacctgcctgacctgcccggc
attgccaacgacctcatgtacattgccgacctgggccccggcattgccccctctgcccct
ggcaccattccagaactgcccaccttccacactgaggtagccgagcctctcaaggtagac
ctacaagatggggtactaaca---------ccacccccaccgcccccaccaccaccccca
gctcctgaggtgctggccagtgcacccccactcccaccctcaaccgcggcccctgtaggc
caaggcgccaggcaggacgacagcagcagcagcgcgtctccttcagtccagggagctccc
agggaagtggttgacccctccggtggctgggccactctgctagagtccatccgccaagct
gggggcatcggcaaggccaagctgcgcagcatgaaggagcgaaagctggagaagaagaag
cagaaggagcaggagcaagtgagagccacgagccaaggtgggcacttgatgtcggatctc
ttcaacaagctggtcatgaggcgcaagggcatctctgggaaaggacctggggctggtgag
gggcccggaggagcctttgcccgcgtgtcagactccatccctcctgtgccgccaccgcag
cagccacaggcagaggaggacgaggacgac

>hyb_panel_chr20_B
gagcagagccggagccaggtgcaggccattggagagaaggtctccttggcccaggccaag
attgagaagatcaagggcagcaagaaggccatcaaggtgttctccagtgccaagtaccct
gctccagagcgcctgcaggaatatggctccatcttcacgggcgcccaggaccctggcctg
cagagacgctcccgccacaggatccagagcaagcaccgcccccctggacgagcgggccctg
caggagaagctgaaggactttcccgtgtgcgtgagcaccaagccggagcccgaggacgat
gcagaagagggacttgggggtcttcccagcaacatcagctctgtcagctccttgctgctc
ttcaacaccaccgagaacctgtacaagaagtatgtcttcctggacccccctggctggtgct
gtaacaaagacccatgtgatgctgggggcagagacagaggagaagctgtttgatgccccc
ttgtccatcagcaagagagagcagctggaacagcaggtcccagagaactacttctatgtg
ccagacctgggccaggtgcctgagattgatgttccatcctacctgcctgacctgcccggc
attgccaacgacctcatgtacattgccgacctgggccccggcattgccccctctgcccct
ggcaccattccagaactgcccaccttccacactgaggtagccgagcctctcaaggcagac
ctacaagatggggtactaaca---------ccacccccaccgcccccaccaccaccccca
gctcctgaggtgctggccagtgcacccccactcccaccctcaaccgcggcccctgtaggc
caaggcgccaggcaggacgacagcagcagcagcgcgtctccttcagtccagggagctccc
agggaagtggtcgacccctccggtggccgggccactctgctagagtccatccgccaagct
gggggcatcggcaaggccaagctgcgcagcatgaaggagcgaaagctggagaagaagaag
cagaaggagcaggagcaagtgagagccacgagccaaggtgggcacttgatgtcggatctc
ttcaacaagctggtcatgaggcgcaagggcatctctgggaaaggacctggggctggtgag
gggcccggaggagcctttgcccgcgtgtcagactccatccctcctctgccgccaccgcag
cagccacaggcagaggaggacgaggacgac

>GM10494.fs20.1
gagcagacccggagccaggtgcaggccattggagagaaggtctccttggcccaggccaag
attgagaagatcaagggcggcaagaaggccatcaaggtgttctccagtgccaagtaccct
gctccagagcgcctgcaggaatatggctccatcttcacgggcgcccaggaccctggcctg
cagagacgctcccgccacaggatccagagcaagcaccgcccccctggacgagcgggccctg
caggagaagctgaagtactttcctgtgtgtgtgagcaccaagccggagcccgaggacgat
```

gcagaagagggacttgggggtcttcccagcaacatcagctctgtcagctccttgctgctc
ttcaacaccaccgagaacctgtacaagaagtatgtcttcctggacccccctggctggtgct
gtaacaaagacccatgtgatgctgggggcagagacagaggagaagctgtttgatgccccc
ttgtccatcagcaagagagagcagctggaacagcaggtcccagagaactacttctatgtg
ccagacctgggccaggtgcctgagattgatgttccgtcctacctgcctgacctgcccggc
attgccaacgacctcatgtacagtgccgacctgggccccggcattgcccccctctgcccct
ggcaccattccggaactgcccaccttccacactgaggtagccgagcctctcaaggcagac
ctacaagatggggtactaacagcaccaccaccacccccaccgcccccaccacctcccccca
gctcctgaggtgctggccagtgcatccccactcccaccctcaaccgcggcccctgtaggc
caaggcgccaggcaggacgacggcagcagcagcgcgtctccttcagtccagggagctccc
agggaagtggtcgacccctccggtggccgggccactctgctagagtccatccgccaagct
gggggcatcggcaaggccaagctgcgcagcgtgaaggagcgaaagctggagaagaagaag
cagaaggagcaggagcaagtgagagccacgagccaaggtggggacttgatgtcggatctc
ttcaacaagctggtcatgaggcgcaagggcatctctgggaaaggacctggggctggtgag
gggcccggaggagcctttgcccgcgtgtcagactccatccctcctctgccgccaccgcag
cagccacaggcagaggaggacgaggacgac

>GM07791.fs20.1
gagcagagccggagccaggtgcaggccattggagagaaggtctccttggcccaggccaag
attgagaagatcaagggcagcaagaaggccatcaaggtgttctccagtgccaagtaccct
gctccagagcgcctgcaggaatatggctccatcttcacgggcgcccaggaccctggcctg
cagagacgctcccgccacaggatccagagcaagcaccgccccctggacgagcgggccctg
caggagaagctgaagtactttcctgtgtgtgtgagcaccaagccggagcccgaggacgat
gcagaagagggacttgggggtcttcccagcaacatcagctctgtcagctccttgctgctc
ttcaacaccaccgagaacctgtacaagaagtatgtcttcctggacccccctggctggtgct
gtaacaaagacccatgtgatgctgggggcagagacagaggagaagctgtttgatgccccc
ttgtccatcagcaagagagagcagctggaacagcaggtcccagagaactacttctatgtg
ccagacctgggccaggtgcctgagattgatgttccgtcctacctgcctgacctgcccggc
attgccaacgacctcatgtacagtgccgacctgggccccggcattgcccccctctgcccct
ggcaccattccggaactgcccaccttccacactgaggtagccgagcctctcaaggcagac
ctacaagatggggtactaacagcaccaccaccacccccaccgcccccaccacctcccccca
gctcctgaggtgctggccagtgcatccccactcccaccctcaaccgcggcccctgtaggc
caaggcgccaggcaggacgacggcagcagcagcgcgtctccttcagtccagggagctccc
agggaagtggtcgacccctccggtggccgggcctctctgctagagtccatccgccaagct
gggggcatcggcaaggccaagctgcgcagcgtgaaggagcgaaagctggagaagaagaag
cagaaggagcaggagcaagtgagagccacgagccaaggtggggacttgatgtcggatctc
ttcaacaagctggtcatgaggcgcaagggcatctctgggaaaggacctggggctggtgag
gggcccggaggagcctttgcccgcgtgtcagactccatccctcctctgccgccaccgcag
cagccacaggcagaggaggacgaggacgac

>BC048328
gagcagagccggagccaggtgcaggccattggagagaaggtctccttggcccaggccaag
attgagaagatcaagggcagcaagaaggccatcaaggtgttctccagtgccaagtaccct
gctccagagcgcctgcaggaatatggctccatcttcacgggcgcccaggaccctggcctg
cagagacgctcccgccacaggatccagagcaagcaccgccccctggacgagcgggccctg
caggagaagctgaagtactttcctgtgtgtgtgagcaccaagccggagcccgaggacgat

gcagaagagggacttgggggtcttcccagcaacatcagctctgtcagctccttgctgctc
ttcaacaccaccgagaacctgtacaagaagtatgtcttcctggacccccctggctggtgct
gtaacaaagacccatgtgatgctgggggcagagacagaggagaagctgtttgatgccccc
ttgtccatcagcaagagagagcagctggaacagcaggtcccagagaactacttctatgtg
ccagacctgggccaggtgcctgagattgatgttccgtcctacctgcctgacctgcccggc
attgccaacgacctcatgtacagtgccgacctgggccccggcattgcccccctctgcccct
ggcaccattccggaactgcccaccttccacactgaggtagccgagcctctcaaggcagac
ctacaagatggggtactaacagcaccaccaccacccccaccgcccccaccacctcccccca
gctcctgaggtgctggccagtgcatccccactcccaccctcaaccgcggcccctgtaggc
caaggcgccaggcaggacgacagcagcagcagcgcgtctccttcagtccagggagctccc
agggaagtggtcgacccctccggtggccgggccactctgctagagtccatccgccaagct
gggggcatcggcaaggccaagctgcgcagcgtgaaggagcgaaagctggagaagaagaag
cagaaggagcaggagcaagtgagagccacgagccaaggtggggacttgatgtcggatctc
ttcaacaagctggtcatgaggcgcaagggcatctctgggaaaggacctggggctggtgag
gggcccggaggagcctttgcccgcgtgtcagactccatccctcctctgccgccaccgcag
cagccacaggcagaggaggacgaggacgac
