# Supplementary material for: Human Subtelomeric WASH Genes Encode a New Subclass of the WASP Family
Source: PLoS Genet. 2007 Dec 21;3(12):e237. doi: 10.1371/journal.pgen.0030237 (PMC2151093; doi:10.1371/journal.pgen.0030237)
Supplement: Supplementary file 2 [file pgen.0030237.sg001.pdf]

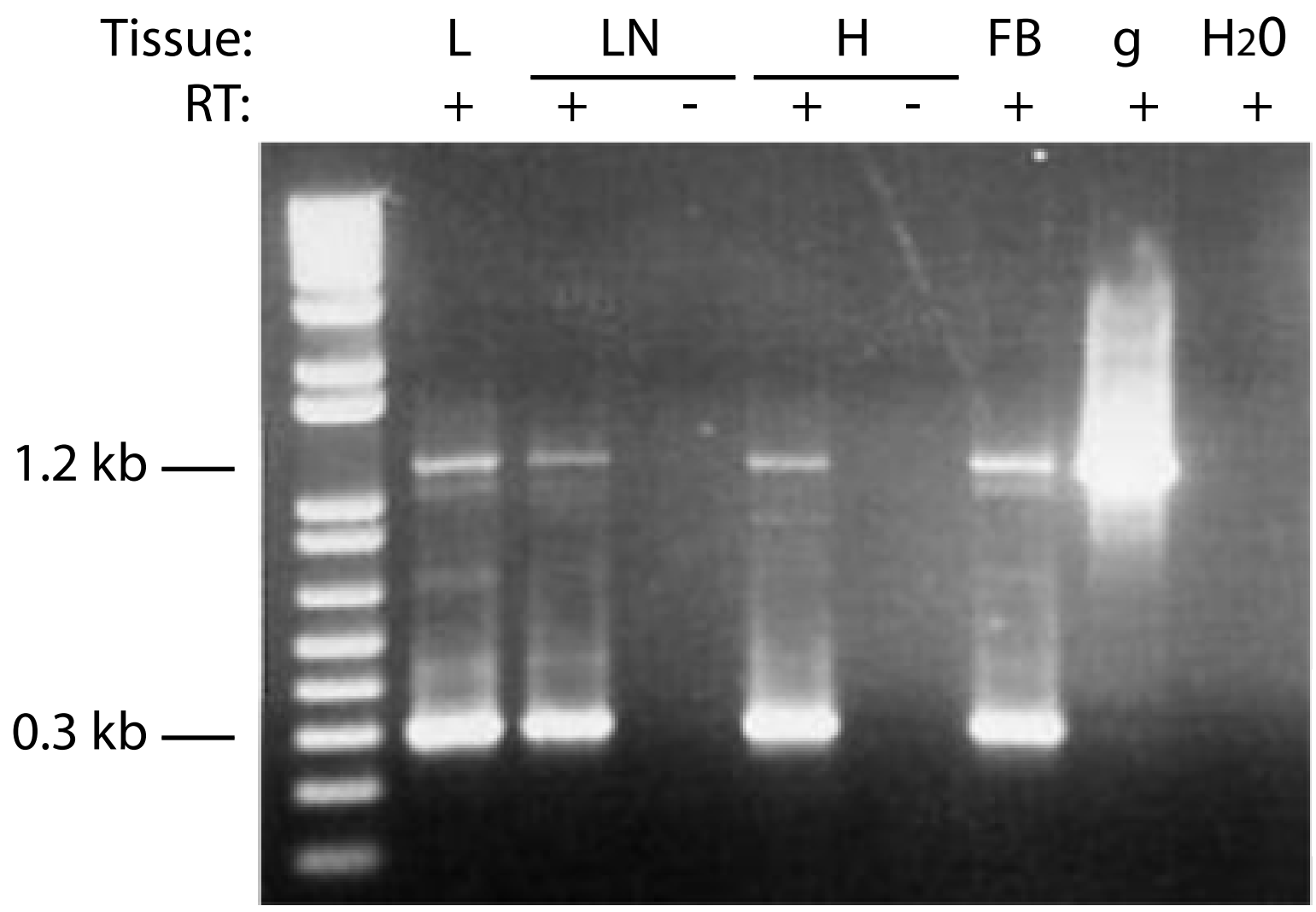

Supplementary Figure S1. RT-PCR confirmation of human WASH expression using exon 8 and exon 10 primers. L, liver; LN, lymph node; H, heart; FB, fetal brain; g, genomic DNA. Reverse-transcriptase (RT)-negative controls were also performed for some tissues. The 0.3-kb band is the expected size for fully spliced WASH transcript.
