# Supplementary material for: Human Subtelomeric WASH Genes Encode a New Subclass of the WASP Family
Source: PLoS Genet. 2007 Dec 21;3(12):e237. doi: 10.1371/journal.pgen.0030237 (PMC2151093; doi:10.1371/journal.pgen.0030237)
Supplement: Supplementary file 3 [file pgen.0030237.sg002.pdf]

A

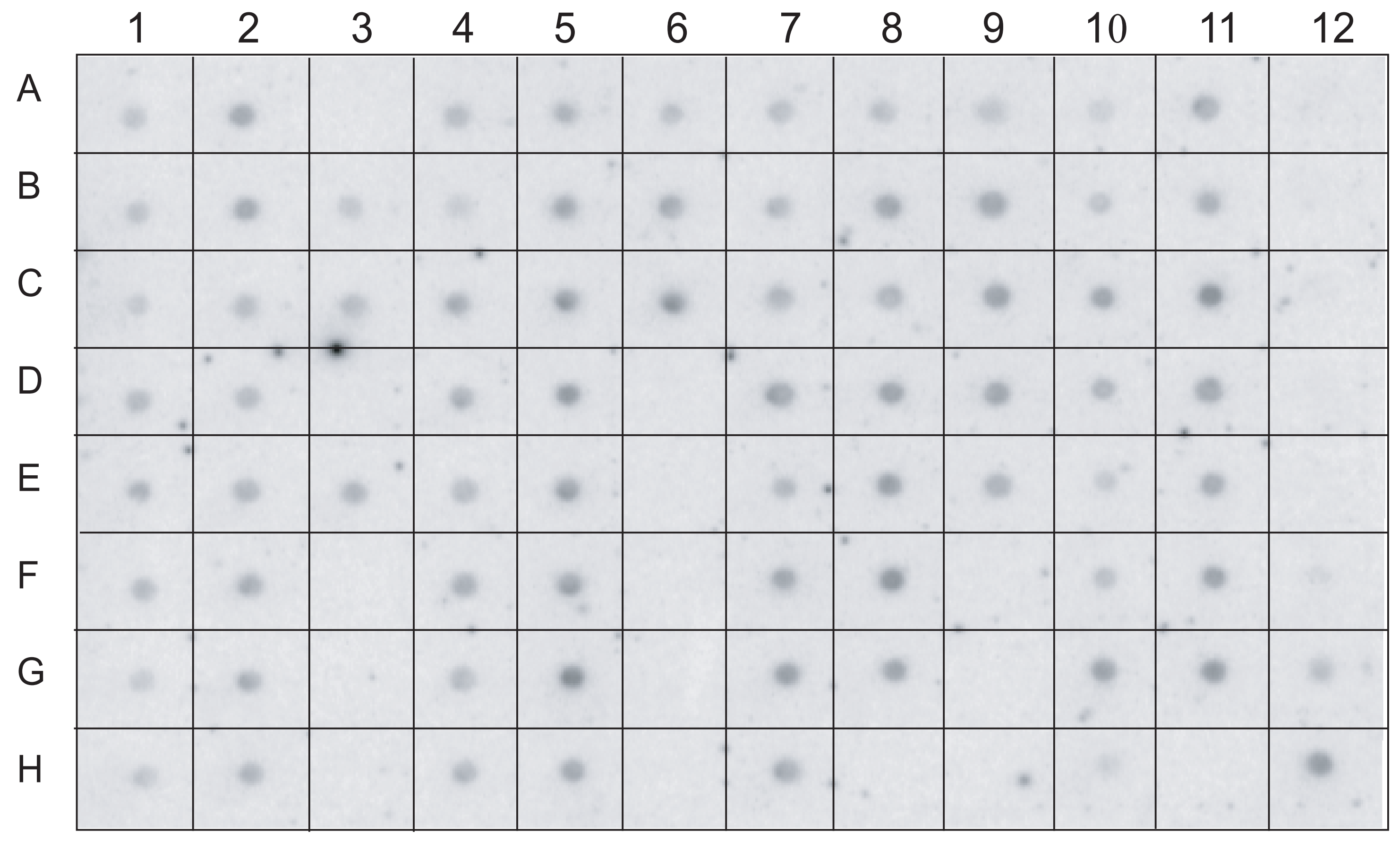

B

	1	2	3	4	5	6	7	8	9	10	11	12
A	whole brain	cerebellum left		heart	esophagus	colon transverse	kidney	lung	liver	leukemia HL60	fetal brain	yeast total RNA
B	cerebral cortex	cerebellum right	accum-bens nucleus	aorta	stomach	colon descending	skeletal muslce	placenta	pancreas	HeLa S3	fetal heart	yeast tRNA
C	frontal lobe	corpus callosum	thalamus	atrium left	duodenum	rectum	spleen	bladder	adrenal gland	leukemia K562	fetal kidney	E.coli rRNA
D	paietal lobe	amygdala		atrium right	jejunum		thymus	uterus	thyroid gland	leukemia MOLT4	fetal liver	E.coli DNA
E	occipital lobe	caudate nucleus	spinal cord	ventricle left	ileum		peripheral blood leukocyte	prostate	salivary gland	Burkitt's lymphoma Raji	fetal spleen	poly r(A)
F	temporal lobe	hippo-campus		ventricle right	ileocecum		lymph node	testis		Burkitt's lymphoma Daudi	fetal thymus	human Cot1 DNA
G	p.g.* of cerebral cortex	medulla oblongata		inter-ventricular septum	appendix		bone marrow	ovary		colorectal adeno-carcinoma SW480	fetal lung	human DNA 100 ng
H	pons	putamen		apex of the heart	colon descending		trachea			lung carcinoma A549		human DNA 500 ng

* paracentral gyrus

C

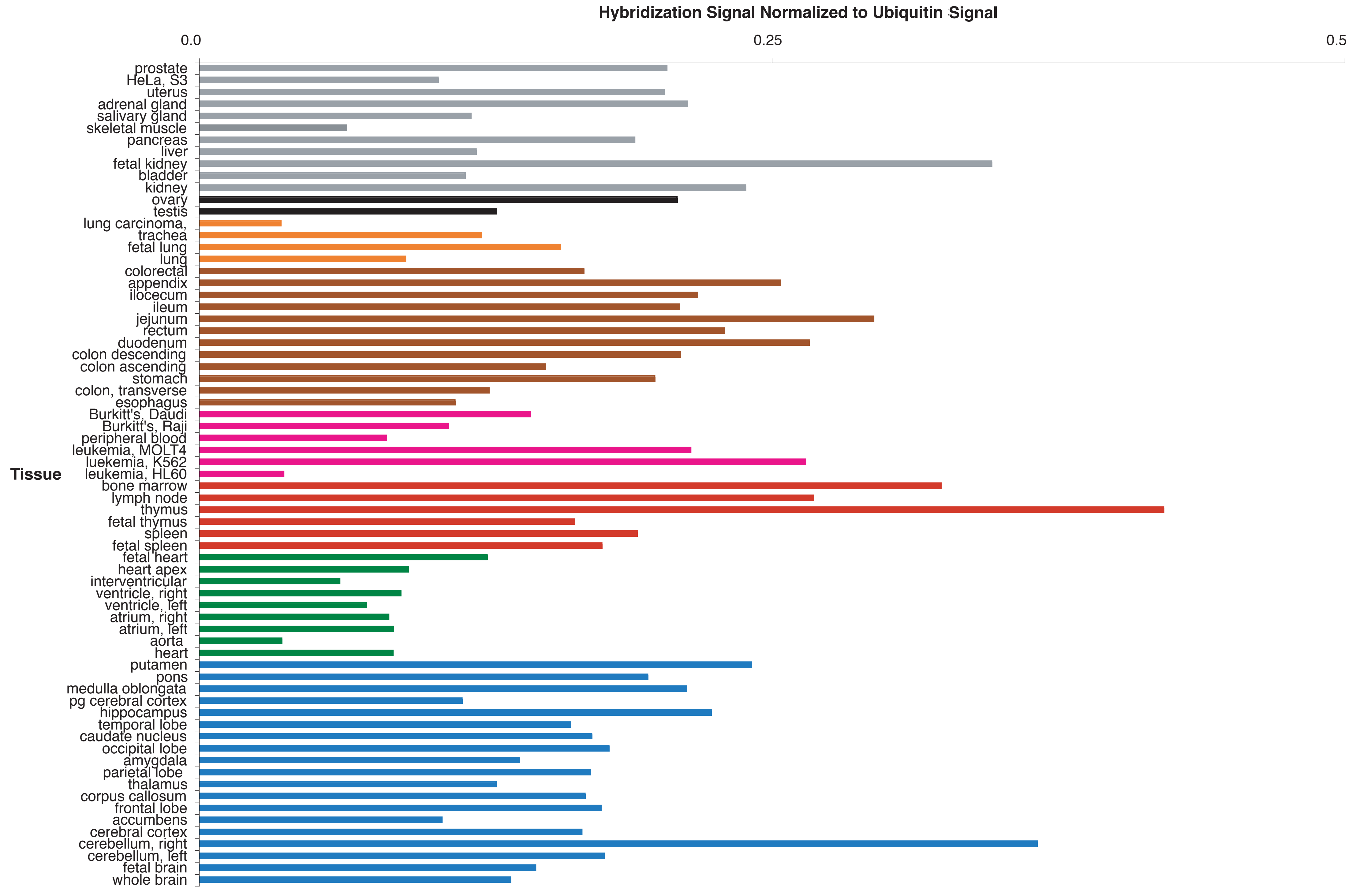

Supplementary Figure S2. Multiple tissue expression array autoradiograph after hybridization using WASH probe (A), the corresponding key to tissues (B), and C) relative WASH hybridization signals normalized to the ubiquitin signal, organized by tissue type.
