# Supplementary material for: Human Subtelomeric WASH Genes Encode a New Subclass of the WASP Family
Source: PLoS Genet. 2007 Dec 21;3(12):e237. doi: 10.1371/journal.pgen.0030237 (PMC2151093; doi:10.1371/journal.pgen.0030237)
Supplement: Supplementary file 4 [file pgen.0030237.sg003.pdf]

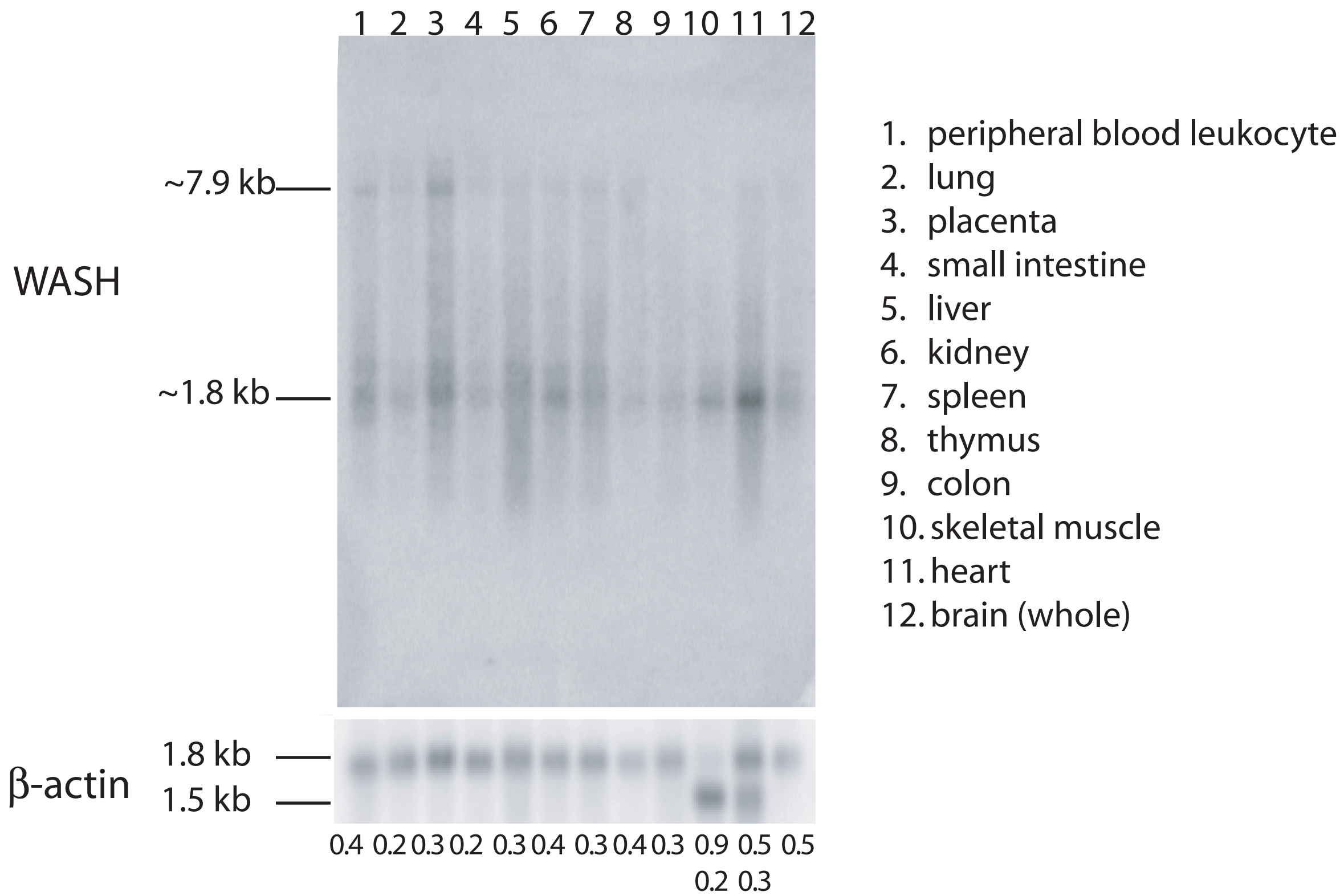

Supplementary Figure S3. Northern blot showing expression of spliced WASH transcripts in multiple human tissues. The ~1.8-kb band corresponds to the expected size of fully spliced, full-length WASH transcripts. The same blot was probed with β-actin as a loading control (bottom). The intensities of the ~1.8-kb WASH band in each tissue are expressed as a fraction of intensity of the β-actin band(s) in the numbers at the bottom of the gel. Where two numbers are given, the top one normalizes to the top actin band, and the bottom normalizes to the sum of the two actin bands.
