# Supplementary material for: Human Subtelomeric WASH Genes Encode a New Subclass of the WASP Family
Source: PLoS Genet. 2007 Dec 21;3(12):e237. doi: 10.1371/journal.pgen.0030237 (PMC2151093; doi:10.1371/journal.pgen.0030237)
Supplement: Supplementary file 5 [file pgen.0030237.sg004.pdf]

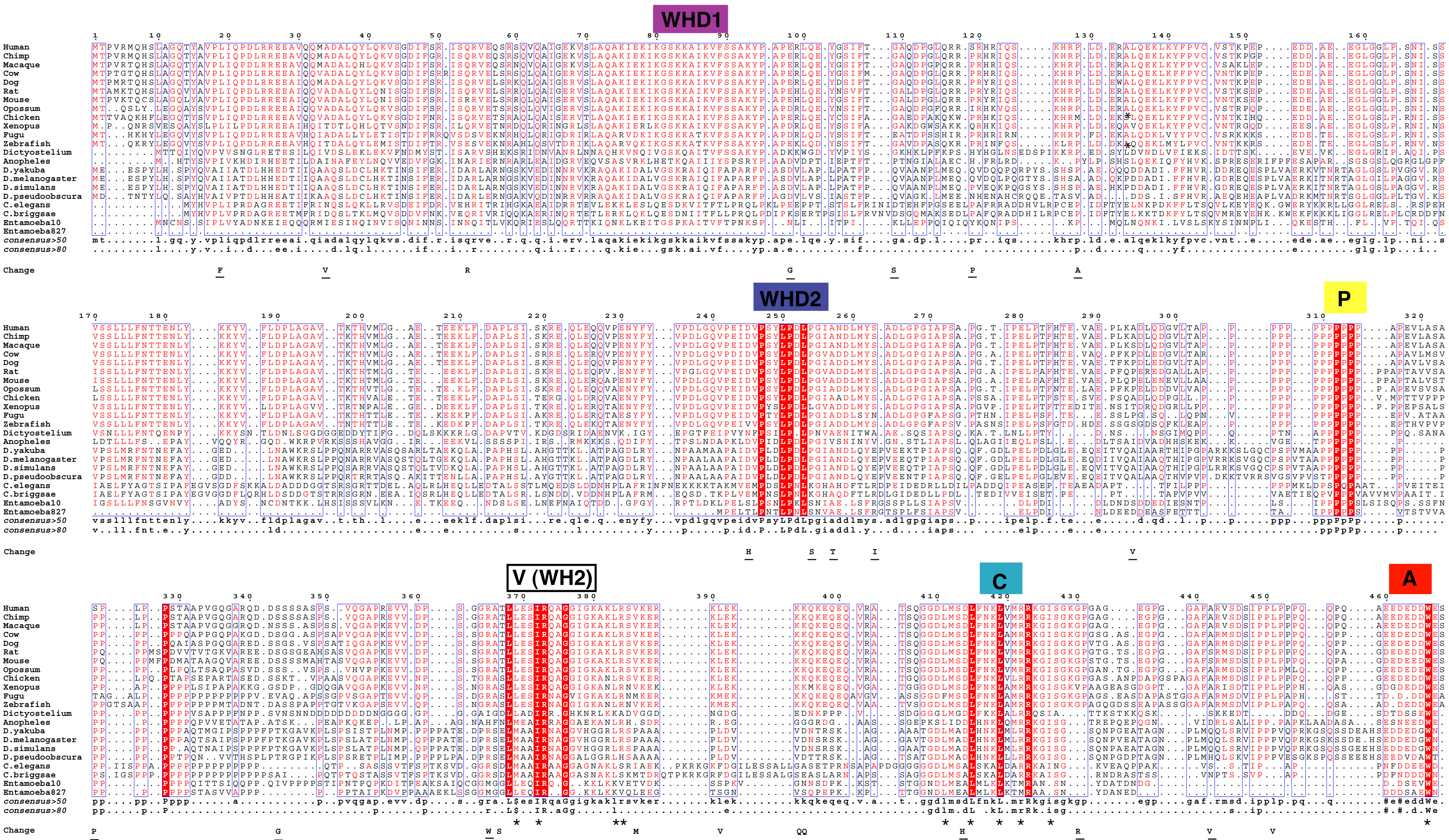

Supplementary Figure S4. Multiple sequence alignment of WASH orthologs. Conserved domains are named above the alignment, using same colors as in Figures 2 and 3A. Boxed residues in red letters are conserved in >50% of sequences, and the >50% consensus indicated at the bottom. Amino acids showing conservation across >80% of the sequences are also shown at the bottom. Amino acids showing 100% conservation are noted against red background. In the consensus sequences, ! is I or V; $ is L or M; % is F or Y, and # is any of NDQEBZ. Asterisks mark conserved residues important for function in known WASP-family members. The line “Change” gives the residues changed by non-synonymous SNPs detected in our survey of human WASH paralogs; underlined changes are non-conservative.
