# Supplementary material for: Human Subtelomeric WASH Genes Encode a New Subclass of the WASP Family
Source: PLoS Genet. 2007 Dec 21;3(12):e237. doi: 10.1371/journal.pgen.0030237 (PMC2151093; doi:10.1371/journal.pgen.0030237)
Supplement: Supplementary file 6 [file pgen.0030237.sg005.pdf]

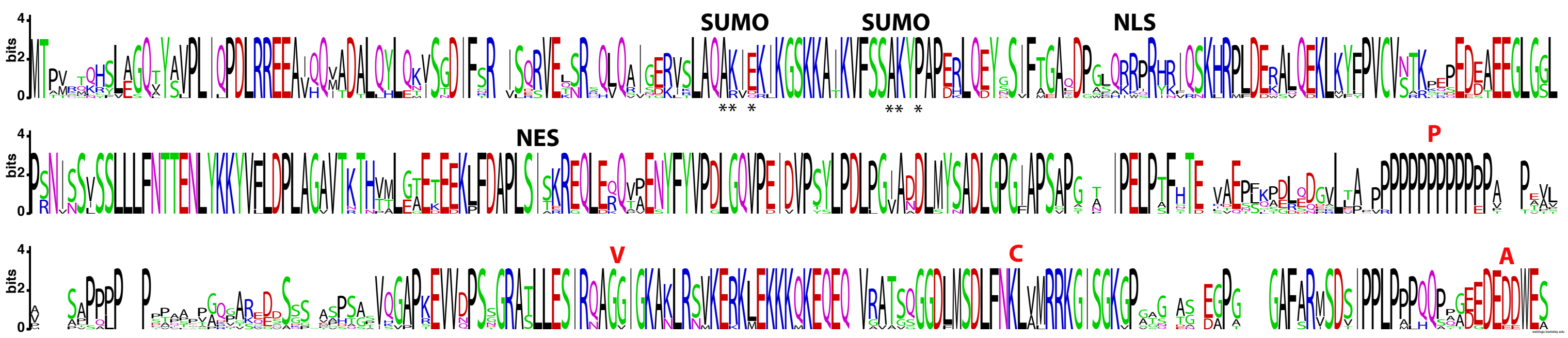

Supplementary Figure S5. Logo plot of vertebrate WASH based on alignment provided in Supplementary Figure S4. Positions of predicted sumoylation sites and nuclear localization and export signals (SUMO, NLS, NES) are indicated. Conserved domains are named above the alignment, using the same colors as used in Figure 3A; the domains extend beyond the labels. Heights of letters indicate degree of conservation in maximum entropy expressed in bits. Similar amino acids are given the same color; KRH, green; DE, blue; and AVLIPWFM, red.
Gaps in the logo plots are regions with residues in only the minority of sequences.
