# Supplementary material for: Human Subtelomeric WASH Genes Encode a New Subclass of the WASP Family
Source: PLoS Genet. 2007 Dec 21;3(12):e237. doi: 10.1371/journal.pgen.0030237 (PMC2151093; doi:10.1371/journal.pgen.0030237)
Supplement: Supplementary file 7 [file pgen.0030237.sg006.pdf]

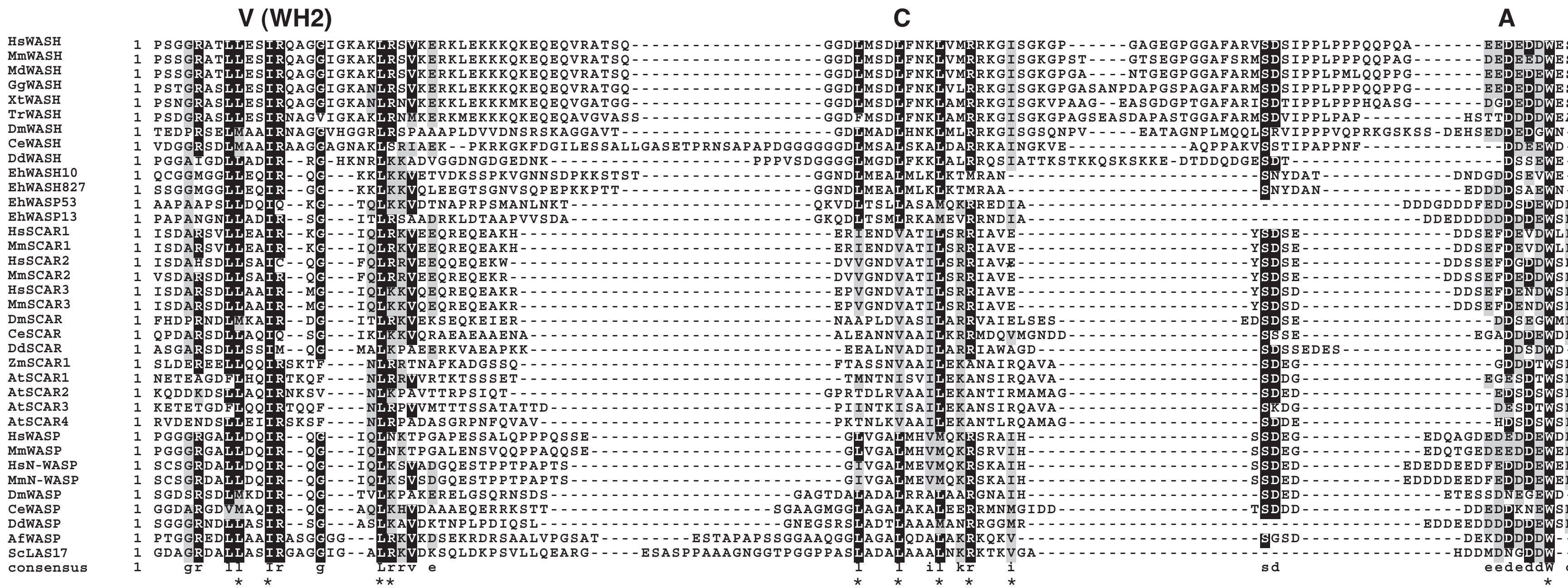

Supplementary Figure S6. Multiple sequence alignment of the VCA region in the C-terminal portion of WASP family members. See Supplementary Figure S4 for details. Species are indicated using two-letter abbreviation before each protein name; the code is provided in Supplementary Information (note: Mm is *Mus musculus*, not *Macaca mulatta*).
