# Supplementary material for: Human Subtelomeric WASH Genes Encode a New Subclass of the WASP Family
Source: PLoS Genet. 2007 Dec 21;3(12):e237. doi: 10.1371/journal.pgen.0030237 (PMC2151093; doi:10.1371/journal.pgen.0030237)
Supplement: Supplementary file 10 [file pgen.0030237.sg009.pdf]

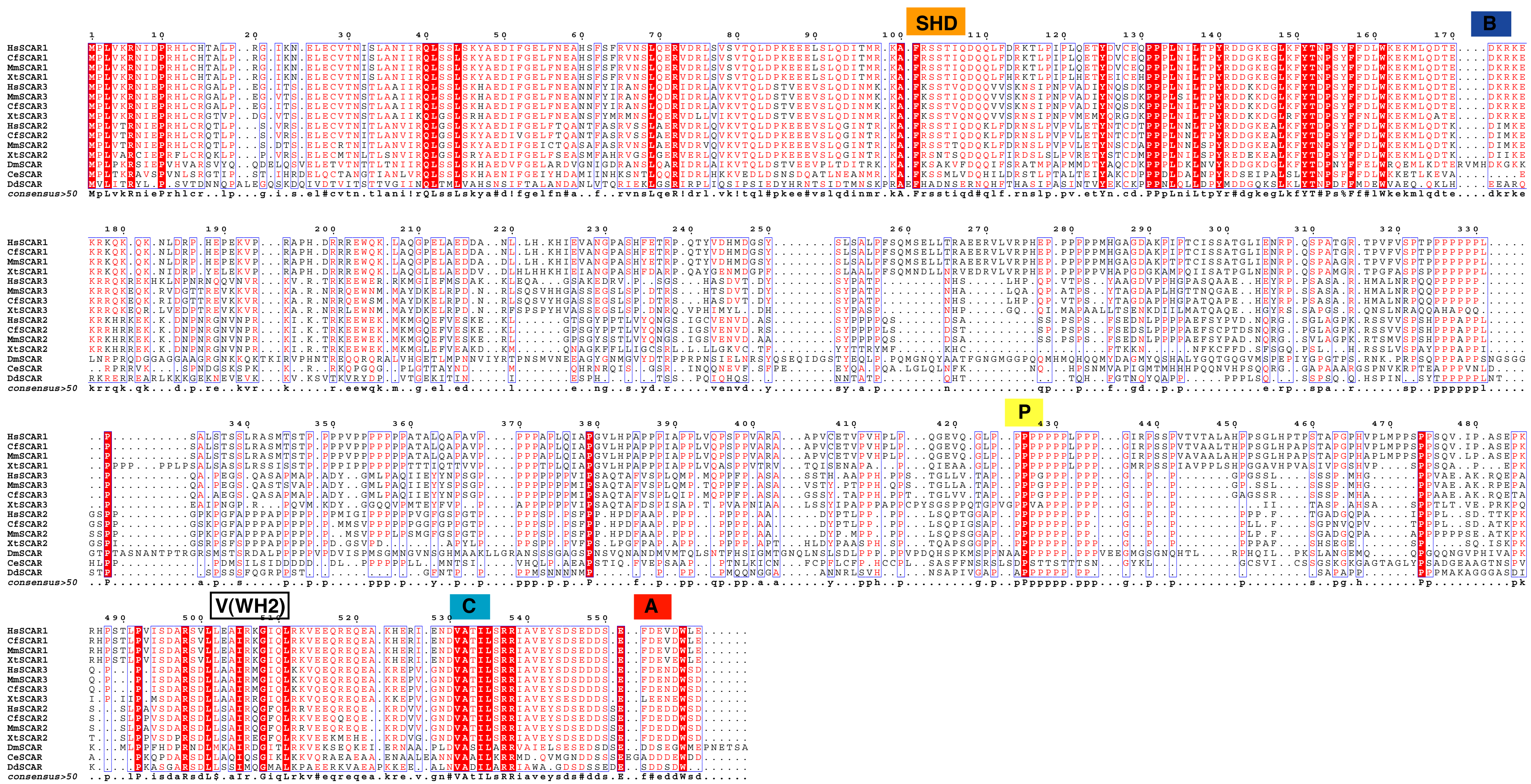

Supplementary Figure S9. Multiple alignment of SCAR (WAVE) orthologs from diverse species. See Supplementary Figure S4 legend for details.
