# Supplementary material for: Human Subtelomeric WASH Genes Encode a New Subclass of the WASP Family
Source: PLoS Genet. 2007 Dec 21;3(12):e237. doi: 10.1371/journal.pgen.0030237 (PMC2151093; doi:10.1371/journal.pgen.0030237)
Supplement: Supplementary file 11 [file pgen.0030237.sg010.pdf]

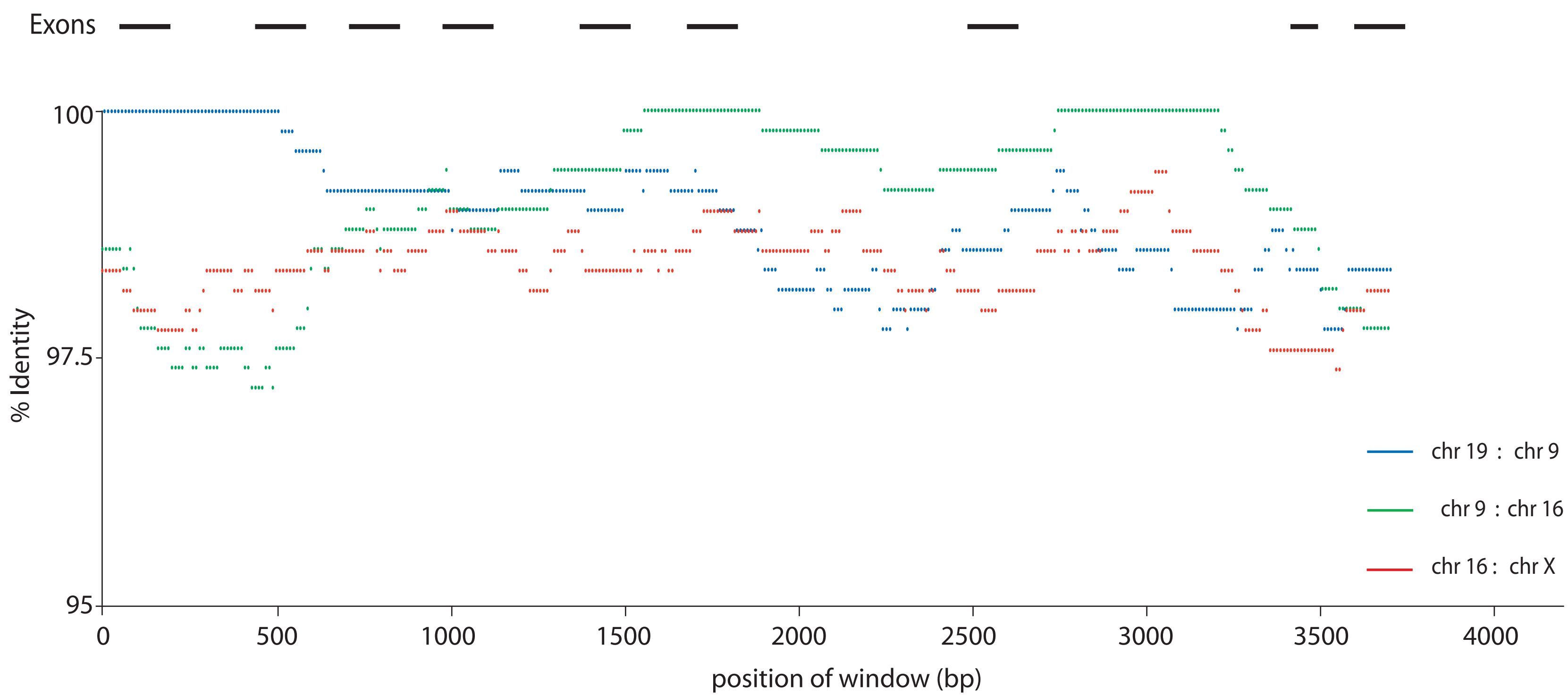

Supplementary Figure S10. Percent identity plots for pairs of WASH genomic sequences that have appear to have under-gone recent sequence exchange (chromosomes 1 vs. 9 and 9 vs. 16, blue and green lines, respectively). The plot for chromosome 16 vs. X plot (red lines) is shown for comparison. Percent identity is plotted in 500-bp windows moving in 10-bp increments. Exchange events appear as shifts to very high sequence similarity. These events are also detected by GeneConv (ref 7 in Supplementary Information), with corresponding Bonferroni-corrected Karlin-Altschul P-values of 0.0016 (g1 setting) and 0.0065 (g0 setting). Position 0 is in intron 1 of WASH, 110 nt preceding the start of 2nd coding exon, corresponding to the start of the partial duplication on 16 and X/Y. The end of the region shown is at nt position 4207 in intron 10.
