# Supplementary material for: Human Subtelomeric WASH Genes Encode a New Subclass of the WASP Family
Source: PLoS Genet. 2007 Dec 21;3(12):e237. doi: 10.1371/journal.pgen.0030237 (PMC2151093; doi:10.1371/journal.pgen.0030237)
Supplement: Supplementary file 12 [file pgen.0030237.st001.pdf]

| HAPLOTYPE SOURCE | # CLONES/ # TOTAL CLONES SEQ'D | | | | | | | | | | | | | | | | | | | | | | | | |
|---|---|---|---|---|---|---|---|---|---|---|---|---|---|---|---|---|---|---|---|---|---|---|---|---|---|
| | | EXON | 1 | | 2 | 3 | | | | | | 4 | | | | 6 | | | | | | | | | |
| | | SEQUENCE VARIANT | C/T | C/T | A/G | A/G | G/C | G/A | G/A | T/C | C/G | T/G/* | T/C | T/C | C/T | T/C | G/C | G/A | C/T | C/T | G/A | C/T | G/T | G/A | C/T |
| | | AMINO ACID | L/F | A/V | Q/R | E/G | T | G/S | G/D | S/P | P/A | Y/D/* | P | C | V | I | D/H | P | P/S | P | A/T | N | S/I | P | A/V |
| | | GENOMIC CO-ORD | 163 | 209 | 6632 | 6972 | 7002 | 7003 | 7004 | 7033 | 7059 | 7276 | 7284 | 7290 | 7365 | 8003 | 8004 | 8012 | 8031 | 8033 | 8040 | 8045 | 8059 | 8108 | 8152 |
| | | VARIATION NO. | 1 | 2 | 3 | 4 | 5 | 6 | 7 | 8 | 9 | 10 | 11 | 12 | 13 | 14 | 15 | 16 | 17 | 18 | 19 | 20 | 21 | 22 | 23 |
| BC048328 | n.a. | | C | C | G | A | G | G | G | T | C | T | T | T | C | T | G | G | C | C | G | C | G | G | C |
| CHR 20 HYB PANEL A | n.a. | | T | - | - | - | - | - | - | - | - | - | - | - | - | - | - | - | - | - | - | - | - | - | - |
| CHR 20 HYB PANEL B | n.a. | | T | T | - | - | - | - | - | - | - | G | C | C | - | - | - | A | - | - | - | - | T | A | - |
| CHR 9 ASSEMBLY | n.a. | | T | - | A | G | - | - | - | C | - | G | - | C | - | C | C | A | - | - | - | - | - | A | T |
| GM10540.genomic.1 | 1/15 | | n.d. | n.d. | n.d. | - | C | A | - | C | - | - | - | C | - | - | - | A | T | T | - | T | T | A | T |
| GM10540.genomic.2* | 2/15 | | n.d. | n.d. | n.d. | - | C | A | - | C | - | G | - | C | - | - | - | A | T | T | - | T | T | A | T |
| GM10540.fs9-12.1 | 2/4 | | n.d. | n.d. | n.d. | - | C | - | - | C | - | G | - | C | - | - | - | A | T | - | - | T | T | A | T |
| GM10540.fs20.1* | 3/3 | | n.d. | n.d. | n.d. | - | C | A | - | C | - | G | - | C | - | - | - | A | T | T | - | T | T | A | T |
| GM07791.fs9-12.1 | 3/3 | | n.d. | n.d. | n.d. | - | C | - | - | C | - | G | - | C | T | - | - | A | T | - | - | T | T | A | T |
| GM07791.fs20.1 | 7/7 | | n.d. | n.d. | n.d. | - | - | - | - | - | - | - | - | - | - | - | - | - | - | - | - | - | - | - | - |
| GM10494.genomic.1 | 1/10 | | n.d. | n.d. | n.d. | - | C | - | - | - | - | G | - | C | - | - | C | A | - | - | A | T | T | A | T |
| GM10494.genomic.2 | 1/10 | | n.d. | n.d. | n.d. | - | - | - | - | C | - | G | - | C | - | - | C | A | - | - | - | - | T | A | T |
| GM10494.genomic.3 | 1/10 | | n.d. | n.d. | n.d. | - | C | - | A | C | G | G | - | C | - | - | - | A | - | - | - | - | T | A | T |
| GM10494.fs9-12.1 | 2/6 | | n.d. | n.d. | n.d. | - | C | - | - | C | - | G | - | C | - | - | - | A | T | - | - | T | T | A | T |
| GM10494.fs20.1 | 7/9 | | n.d. | n.d. | n.d. | - | - | - | - | - | - | - | - | - | - | - | - | - | - | - | - | - | - | - | - |

**Supplementary Table 1**. Locations of sequence variants identified in 14 different WASH ORFs that appear to be intact, at least in coding exons 2-10. We used the cDNA sequence BC048328 as reference; "-" indicates where a sequence is not different from BC048328. These variants were derived from chromosomes captured in a monochromosomal hybrid panel, certain chromosomes isolated by flow-sorting (20 or 9-12) from three individuals, and copies PCR amplified from genomic DNA (without chromosomal assignment) from the same individuals. Note: Two ORFs in one individual (with asterisks) were identical in coding sequence, but differed by 3% in intronic sequence implying that they derive from different chromosomal locations. n.a., not applicable; n.d., not determined. The SNP highlighted in blue is unconfirmed, as it was observed only once in this survey and is not a variant site in EST sequences in public databases. The variant sequences are provided in fasta format in Supplementary Data File.

| 7 | | | | | 8 | | | | | | | 9 | 10 | | | | | | |
|---|---|---|---|---|---|---|---|---|---|---|---|---|---|---|---|---|---|---|---|
| 9nt/del | T/A | T/C | A/G | G/A | C/T | C/T | G/A | G/A | C/G | A/C | A/C | G/C | G/A | C/T | C/T | C/G/T | G/A | C/T | G/C |
| APP/del | P | P/S | S/G | A | V | R/W | G/S | V/M | L/V | K/Q | K/Q | D/H | G/R | A/V | R | L/V/L | V | D | S |
| 8270 | 8289 | 8320 | 8377 | 8391 | 9086 | 9102 | 9144 | 9165 | 9180 | 9189 | 9192 | 9998 | 10198 | 10235 | 10239 | 10261 | 10263 | 10302 | 10314 |
| 24 | 25 | 26 | 27 | 28 | 29 | 30 | 31 | 32 | 33 | 34 | 35 | 36 | 37 | 38 | 39 | 40 | 41 | 42 | 43 |
| 9nt | T | T | A | G | C | C | G | G | C | A | A | G | G | C | C | C | G | C | G |
| - | - | - | - | - | - | - | - | - | - | - | - | - | - | - | - | - | - | - | - |
| del | A | C | - | - | - | T | - | A | - | - | - | C | - | - | - | - | - | - | C |
| del | A | C | - | A | - | T | - | A | - | C | C | C | - | T | - | T | - | - | - |
| del | A | C | - | - | - | T | A | A | - | - | C | C | - | - | - | - | - | - | n.d. |
| del | A | C | - | - | - | T | A | A | - | - | C | C | - | - | - | - | - | - | n.d. |
| del | A | - | - | - | - | T | - | A | G | - | - | C | A | - | - | - | - | - | n.d. |
| del | A | C | - | - | - | T | A | A | - | - | C | C | - | - | - | - | - | - | n.d. |
| del | A | - | G | - | - | T | - | A | G | - | - | C | A | - | - | - | - | - | n.d. |
| - | - | - | G | - | - | - | - | - | - | - | - | - | - | - | - | - | - | - | n.d. |
| del | A | C | - | - | - | T | - | A | G | - | - | C | A | - | - | G | - | C | n.d. |
| del | A | C | - | - | T | T | - | A | - | - | - | C | - | - | - | G | - | - | n.d. |
| del | A | C | - | A | - | - | - | A | G | C | C | C | - | - | - | - | - | - | n.d. |
| del | A | C | - | - | - | T | - | A | G | - | - | C | A | - | T | - | A | - | n.d. |
| - | - | - | - | - | - | - | - | - | - | - | - | - | - | - | - | - | - | - | n.d. |

Synonymous Substitutions

Non-Synonymous Substitutions

Stop/Frameshift Mutations

SNPs without support
