# Supplementary material for: Human Subtelomeric WASH Genes Encode a New Subclass of the WASP Family
Source: PLoS Genet. 2007 Dec 21;3(12):e237. doi: 10.1371/journal.pgen.0030237 (PMC2151093; doi:10.1371/journal.pgen.0030237)
Supplement: Supplementary file 13 [file pgen.0030237.sd002.doc]

**Supplementary Information**

Linardopoulou et al.

**METHODS**

**Genomic sequence analysis**

Copies of the WASH gene in the March 2006 human (Hs) genome assembly and additional paralogs and alleles we sequenced (see below) were analyzed for the presence of a full-length open reading frame (ORF) by comparison to the ORF in the BC048328 cDNA using the WISE 2 program^1^ (http://www.ebi.ac.uk/Wise2/). This program uses a protein sequence as a template to predict a related protein sequence in the analyzed genomic region. This prediction is based on protein similarity searches using three frame translations of the analyzed genomic region and virtual splicing of the DNA sequence at consensus splice sites.

To obtain sequences of WASH orthologs in other species, we used *Mus musculus* (Mm) orf19 (XP_128709.2), *Drosophila melanogaster* (Dm) CG13176 (NP_610739.1) and *Caenorhabditis elegans* (Ce) Y48E1B.1 (NP_496847.1) sequences to search the non-redundant protein database at NCBI (http://www.ncbi.nlm.nih.gov/blast/) with no filter and expect parameter of 1000. The following protein sequences were obtained: *Pan troglodytes* XP_001145900, *Bos taurus* XP_613061.2, *Canis familiaris* (Cf) XP_543873.2, *Rattus norvegicus* XP_346084.2, *Gallus gallus* (Gg) NP_001006245, *Xenopus tropicalis* (Xt) NP_001016854.1, *Danio rerio* XP_682788.1, *Drosophila pseudoobscura* EAL24940.1, *Caenorhabditis briggsae* CAE73332.1, *Dictyostelium discoideum* (Dd) XP_629407, and *Entamoeba histolytica* (Eh) XP_647908.1 (827.t00001, EhWASH1) and XP_656631.1 (10.t00024, EhWASH2). We also used the same three protein sequences as queries for BLAT searches of different genomes available through the UCSC Genome Browser (http://genome.ucsc.edu/). Retrieved matches were used as entries for the Wise 2 program, along with the corresponding query protein sequence, to computationally derive protein sequences. Protein sequences for the following species were obtained using this approach: *Monodelphis domestica* (opossum), *Takifugu* *rubripes* (fugu) (Tr), *Anopheles gambiae* (mosquito), *Drosophila yakuba*, *D. simulans*, and *D. mojavensis*. The protein sequence for *Macaca mulatta* (rhesus macaque) was obtained using *in silico* assembly and translation of sequences in Trace Archive matching the human protein. The computationally derived WASH sequences are provided in fasta format in the Supplementary Data File.

We also performed phylogenetic analyses of known WASP-family members and WASH orthologs from diverse species. We obtained the following protein sequences from the NCBI protein database (http://www.ncbi.nlm.nih.gov/entrez/query.fcgi?db=Protein): *Homo sapiens* (Hs) WASP (P42768), N-WASP (O00401), SCAR1 (Q92558), SCAR2 (Q9Y6W5), and SCAR3 (Q9UPY6); *Mus musculus* (Mm) WASP (P70315), N-WASP (Q91YD9), SCAR1 (Q8R5H6), SCAR2 (Q8BH43), and SCAR3 (Q8VHI6); *Drosophila melanogaster* (Dm) WASp (CG1520), SCAR (CG4636); *Caenorhabditis elegans* (Ce) WASp (Q17795) and WAVE (SCAR) (NP_493028); *Dictyostelium discoideum* (Dd) WASp (AAG24442) and SCAR (AAD29083); *Canis familiaris* (Cf) WASP (XP_548986), SCAR1 (XP_532260.2), SCAR2 (XP_544469.1), SCAR3 (XP_534525.2), *Xenopus tropicalis* (Xt) N-WASP (AAH67309), SCAR1 (AAH91579.1); *Saccharomyces cerevisiae* (Sc) Las17p (NP_014824); and *Aspergillus fumigatus* (Af) WASP (XP_747824.1). SCAR2 and SCAR3 were predicted from the *Xenopus tropicalis* genome, and N-WASP from the *Canis familiaris* genome, using WISE 2 as described above. These computationally derived predicted WASP and SCAR proteins are supplied in fasta format at the end of this document. The following plant SCAR sequences were used in the C-terminal alignment only: *Arabidopsis thaliana* (At) SCAR1 (AAU93851), SCAR2 (AY743924), SCAR3 (AAU93850), and SCAR4 (AAU93852); *Zea mays* (Zm) SCAR1 (BM340311, cDNA). Sequences of *Entamoeba histolytica* (Eh) hypothetical proteins 53.t00004 (XP_654445) and 13.t00006 (XP_656416), which are other WASP-family members, were obtained from the TIGR database (*Entamoeba histolytica* Genome Project http://www.tigr.org/tdb/e2k1/eha1/) by similarity searching with the hypothetical protein 10.t00024 (XP_656631.1) sequence as a query. Sequences are identified in figures by two-letter species codes indicated above in parentheses.

We used the Multalin program (http://prodes.toulouse.inra.fr/

multalin/multalin.html)^2^ to align the protein sequences. The resulting alignments were edited by hand. Logo plots^3^ were generated with WebLogo 3 (reference ^4^, (http://weblogo.berkeley.edu/logo.cgi) from multiple sequence alignments of WASH, WASP, and SCAR orthologs (Supplementary Figures S4, S8, and S9), respectively. The height of each letter in a LogoPlot reflects the degree of conservation of a particular residue among aligned sequences and is the maximum entropy expressed in bits. This value decreases with inclusion of more divergent species in an alignment, unless the residue is conserved. We used the WebLogo3 option allowing for small sample correction, because, when there are only a few sample sequences, a straightforward calculation will tend to overestimate the entropy. To compensate for this, the Small Sample Correction option will subtract an approximation of this bias from the total entropy. This small-sample correction depends only on the number of symbol types (4 for RNA/DNA, 20 for protein) and the total amount of data in each column, which may differ from one column to another, since some columns will contain more gaps, and less data. Positions for which the majority of sequences have a gap in an alignment will appear as blanks on the LogoPlot.

A neighbor-joining phylogenetic tree was constructed from the C-terminal portion of WASP-family members using PAUP* 4.0 (http://paup.csit.fsu.edu/); bootstrap values were calculated over 1000 iterations.

Using K-estimator^5^, we examined an alignment of sequences comprising the ten WASH coding exons from human, macaque, dog, cow, mouse, rate, chicken, and frog for regions subjected to positive selection with a sliding window size of 500 bp moved in 100-bp increments, and 100 bp windows in 50-bp increments.  Sequences from the same set of species except frog for signs of positive selection using Phylogenetic Analysis Using Maximum Likelihood (PAML)^6^.

The genomic sequences in the March 2006 assembly were also analyzed by PlotID (E. Williams and E. Linardopoulou, unpublished) to detect fluctuations in percent identity across pairwise alignments and by GeneConv^7^, a program that tests sequences for patterns of variation that might have arisen from gene-conversion like events (including exchange and double-recombination). Details are provided elsewhere^8^.

**Protein motif predictions**

We searched for known conserved motifs and protein domains in the vertebrate consensus sequence of the WASH protein and compared their locations to regions of interspecies conservation. We used the following prediction programs: NCBI conserved-domain search (http://www.ncbi.nlm.nih.gov/blast/) with Reverse Position-Specific BLAST (rpsblast) ^9^; SMART (simple modular architecture research tool) ^10, 11^ (http://smart.embl.de/); Block searcher^12^ (http://blocks.fhcrc.org/blocks/blocks_search.html); PSORT (protein localization site prediction)^13^ (http://psort.nibb.ac.jp/); sumoylation signal prediction (SUMOplot) ^14^ (<http://www.abgent.com/doc/sumoplot>); and nuclear export signal prediction NESnet^15^ (<http://www.cbs.dtu.dk/services/NetNES/>). Our search of the NCBI Conserved Domain databases using rpsblast without the default filter of low complexity regions revealed weak similarity to the Wiskott-Aldrich syndrome protein (WASP) family (29% identity to KOG3671 domain). The SMART program identified the same region as a predicted actin-binding WH2 domain, albeit with an E-value (1.82e+01) below the required threshold. A BLOCKS database search revealed significant similarity to a family of actin-binding WH2 domain-containing proteins, including the yeast WASP ortholog Las17 (blocks IPB003124A-B (2/2) with a combined E-value of 6.8e-09).

**WASH transient expression and actin co-localization**

Since other WASP family members have been shown to co-localize with actin in COS-7 cells (e.g., ^16^), we transiently over-expressed GFP-WASH in COS-7 cells in which actin was stained with phalloidin and nuclei with DAPI. Full-length WASH derived from the BC048328 cDNA was cloned in the BglII site of pEGFP-N1 vector from Clontech Inc. (Mountain View, CA) such that the GFP is added to the C-terminus of WASH. African Green Monkey kidney fibroblast COS-7 cells were transfected with plasmid (2 µg) for 5 h without serum using Lipofectamine 2000 (Invitrogen, Carlsbad, CA) according to Invitrogen’s instructions. The cells were induced to divide with 10% fetal bovine serum (Invitrogen) overnight and checked for GFP expression the following day. The cells were then plated onto 0.1% gelatin-coated 8-chambered slide, grown to achieve ~30% confluence, fixed with 4% paraformaldehyde (Sigma Aldrich, St. Louis, MO) in PBS for 15 min at room temperature, washed 3 times in PBS, permeabilized with chilled 0.1% Triton X-100 (Sigma Aldrich, MO) in PBS for 10 min at room temperature in a humid chamber, and washed again 3 times in PBS. Cells were blocked in DMEM + 10% FBS for 1 h at room temperature in a humid chamber. Excess fluid was blotted off, and actin was labeled by incubating the cells in either a 1:1000 fold dilution of Texas Red-conjugated phalloidin (Sigma Aldrich) or a 1:300 fold dilution of Texas Red-conjugated phalloidin (Molecular Probes) in PBS + 5% goat serum for 45 min at room temperature in humid chamber. The cells were washed three times in PBS for 3 min each. Nuclei were labeled with 0.1 µg/ml DAPI in PBS + 20% Tween-20 (Sigma Aldrich) and washed three times with PBS + 0.1% Tween 20 and three times with PBS before mounting. The slides were mounted in Vectashield (Vector Labs, Burlingame, CA) and imaged using a laser-scanning confocal microscope (Zeiss, Thornwood, NY). Images were pseudocolored and overlaid using Image J (http://rsb.info.nih.gov/ij/).

**Pyrene actin-polymerization assays**

Our actin-polymerizatin assays used WASH VCA (amino acids 352-468 of the BC048328 open reading frame) and N-WASP VCA (amino acids 398–505 of NM_003941) constructs. These constructs were cloned into a modified pGEX (GE, Piscataway, NJ) vector using standard polymerase chain reaction cloning techniques. The pGEX vector was modified to include a HIS-tag 3’ to the multiple cloning site to allow for expression of double-tagged proteins.

To express these proteins, constructs in the modified pGEX vector (described above) were transformed into the Rosetta–Gami B BL21(DE3) pLysS strain (Novagen). Protein induction and bacterial lysate preparation were performed as previously described^17^. Lysates were first coupled to Fastflow nickel-sepharose 6 (GE) by incubation for 3 h at 4°C, washed as previously described^17^ using T100G5 buffer (50 mM Tris, pH 7.6, 100 mM NaCl, 5 mM DTT, 5% glycerol) with 50 mM imidazole and eluted according to manufacturer’s instructions using T100G5 buffer with 400 mM imidazole. His elutions were diluted with T100G5 buffer and coupled to glutathione-sepharose 4B (GE) by incubation for 3 h at 4°C, washed using T100G5 buffer, treated with Precision Protease (GE) according to manufacturer’s instructions, dialyzed into the G buffer (2 mM Tris, pH 8, 0.1 mM CaCl, 0.1 mM DTT, 5% glycerol), concentrated as necessary using Centricon centrifugal filter devices (Millipore), flash frozen in G-Buffer (as above + 0.1 mM ATP) + 50% glycerol, and stored at -80°C.

Pyrene actin-polymerization assays were performed as previously described^17^ using 20 nM Arp2/3 (Cytoskeleton.com). The data were normalized as previously described^17^. To scale the data, the half-maximal value of fluorescence for 100 nM N-WASP VCA + Arp2/3 was set as 1.0 arbitrary units.

***Drosophila* genetics**

Flies were cultured and crossed on yeast-cornmeal-molasses-malt extract medium at 25°C. The CG13176^EY15549^ flies, which carry a P element inserted between nucleotides 29 and 30 of the coding region, are homozygous viable with no apparent phenotype, suggesting that in this line *washout* function is not disrupted. The *washout*[^∆^](http://flybase.bio.indiana.edu/.bin/fbidq.html?FBti0070828)^185^ allele was generated by the imprecise excision of this P element (Figure 6), as previously described ^18^. Sequence analysis of the *washout*[^∆^](http://flybase.bio.indiana.edu/.bin/fbidq.html?FBti0070828)^185^ allele indicates an internal deletion of 1029 nucleotides, which truncates the protein following amino acid 10 (Figure 6). Alleles with precise excisions of the P element, which was confirmed by sequence analysis of one such allele, are homozygous viable, consistent with the presence of an uninterrupted *washout* ORF.

For the lethality test, flies carrying *washout*[^∆^](http://flybase.bio.indiana.edu/.bin/fbidq.html?FBti0070828)^185^ allele were crossed to a balancer chromosome carrying GFP, which allows for easy detection and separation of *wash*^∆185^ homozygous embryos based on the absence of GFP fluorescence.

**Fluorescence in situ hybridization (FISH)**

Metaphase spreads were prepared from the following human cell lines using published methods^19^: GM10494 (Mbuti Pygmy), GM07791 (Caucasian), GM10540 (Melanesian), GM08792 (Caucasian), CGM1, and a primary PHA-stimulated peripheral blood culture of a healthy donor (H1-H5, respectively). All are from males. Cell lines with prefix GM are from the Coriell NIGMS cell repository (http://ccr.coriell.org/ccr/); CGM1 was originally obtained from M. Olson. Non-human primate cell lines were chimpanzee: PT5 (female) obtained from H. Willard and CRL1847 (male) from ATCC (C1 and C2, respectively); gorilla AG05251B (female) from Coriell, orangutan CRL1850 (female) from ATCC, and stump-tailed macaque (Macaque arctoides) GM03443 (female) from Coriell. FISH probes consisted of cloned PCR products that were generated using BAC RP11-395L14, which contains the 2q13-q14 copy of WASH, as template. Primers were situated to avoid large stretches of interspersed repeat DNA (Table A). PCR reactions contained 1x PCR buffer, 2 mM MgCl_2_, 0.5 mM dNTPs, 400 nM each primer, 2.5 units Biolase (Bioline, Randolph MA), and 1 ng BAC DNA. PCR temperature profile was: 2 min at 95°C; 15 cycles of 10s at 94°C, 30s at 62°C, 5 min at 72°C; 15 cycles of 10s at 94°C, 30s at 62°C, 5min + 20s/cycle at 72°C; 10 min at 72°C; hold at 4°C.

| Table A. Primers used to generate FISH probes | | |
| --- | --- | --- |
| Primer | Sequence | Product size (bp) |
| A-gred3F | AAGCCTGGCTGTGTCTTCTC | 5076 |
| A-gred3R | AACTGCCTCCCTTCCTCCT |  |
| B-gred2F | CAAGAGGAAAGAGGTGCCTG | 2963 |
| B-gred2R | GTGGAACTGAGGAGCTAGGG |  |
| C-gred1F | TCTTCTGCCCTTCATCCTTC | 4781 |
| C-gred1R | CAGGCACCTCTTTCCTCTTG |  |
| D-gred5F | CCTCATGACCAGCTTGTTGA | 4877 |
| D-gred5R | GAGGACCCTTTACAGCAGATG |  |
| E-gred4F | CAAATTGCCGGATTTCCTT | 4627 |
| E-gred4R | GCCTGAACAAGTGAGGCTCT |  |

The products were cloned in the TOPO TA vector (Invitrogen, Carlsbad, CA). Plasmid DNA was prepared from bacterial stocks and biotinylated by nick-translation. Probes A-C (the N-terminal portion encompassing 5’UTR and first exon) and A-E (includes distal exons 2-10) were pooled for hybridization to human and non-human chromosomes, respectively. Stump-tailed macaque chromosomes were hybridized with a pool of WASH gene PCR products representing the orthologous locations of probes D and E in the January 2006 rhesus macaque genome assembly: the two primer pairs used to amplify from stump-tailed macaque genomic DNA are listed in Table B.

| Table B. Macaque genomic sequence primers used to generate macaque FISH probes | | | |
| --- | --- | --- | --- |
| Primer | sequence | genomic coordinates  (macaque Jan 2006) | Product size (bp) |
| D-macF | AGAGAGTGCACGGTCCCTG | Chr13:113700042-113704035 | 3994 |
| D-macR | CCCAGCTCACCTGTCTTGAG |  |  |
| E-macF | CTCAAGACAGGTGAGCTGGG | Chr13:113704016-113707971 | 3956 |
| E-macR | AAGGCGCTGGTGGAGAAC |  |  |

FISH probes were hybridized to metaphase preparations and detected with FITC-conjugated avidin, and chromosomes were counterstained with DAPI to generate a cytogenetic banding pattern as described elsewhere^20^.

For each experiment (a given probe on a particular individual), we analyzed five to ten well-labeled, well-spread metaphase cells. Signals were scored at each location on an intensity scale of 1 to 4. In each metaphase, we also noted whether probe signal was present on only one or both homologs at each location. A location was deemed to be heterozygous for the presence of the probe if signal was observed at the location in >50% of cells and signal was observed on both homologs in no more than one cell and the 2^nd^ homolog was given a signal score of only 1 (in which case, 1 was subtracted from the overall score for that location). The sum of the intensity scores at each location was corrected for the number of analyzed chromosomes (usually two times the number of metaphases except X, Y, and locations deemed heterozygous for probe presence, and some cases of missing chromosomes in incomplete spreads). The corrected cumulative scores were then normalized to the maximum cumulative score in the experiment. Figure 7 reports locations in which the normalized cumulative score was ≥20% of the site with maximum signal. Reported polymorphic sites did not reach this detection threshold on one or both homolog in one or more individuals. Locations in non-human primate chromosomes are indicated using the numbering in the human karyotype.

We also mapped the mouse WASH ortholog orf19 by FISH since its genomic location and copy number was uncertain. A 2003 report assigned orf19 to the mouse X chromosome^21^, but orf19 is found on chromosome 17 in the Feb 2006 mouse genome assembly. We used a pool of three orf19-locus probes, each ~4 kb, for FISH. The probes were generated by PCR from mouse genomic DNA using the following primers: mcxy 1F 5’ GGAGGAACCCTTGAGCTGAG 3’, mcxy 1R 5’ GTTACCGGCTCCAAGAAACC 3’, mcxy 2F 5’ ACGATTGGAGGCCAAGGTAG 3’ , mcxy 2R 5’ CTCTCCGCACTGTAGCAACC 3’, mcxy 3F 5’ GGTTGCTACAGTGCGGAGAG 3’, and mcxy 3R 5’ CCCAGAGGGACAAGGATAGC 3’. The PCR conditions were as above for human FISH probes. The probe was labeled and hybridized in the presence of mouse Cot1 DNA to metaphase spreads prepared from lipopolysaccharide-stimulated splenocytes from Bl6 mice as described above. FISH revealed a single internal location on mouse chromosome 17 (not shown), consistent with the placement of orf19 in the mouse genome assembly.

**Gene expression**

The expression profile of human WASH was determined in three ways. First, we hybridized a WASH probe to a Multiple Tissue Expression Array from Clontech Inc. (Mountain View, CA). The probe was the 700-bp insert of a cloned WASH RT-PCR product generated from lymph node using primers BC19(F) 5’ TGACTCCTGTGAGGATGCAG 3’ and chr2(R) 5’ ATGTACATGAGGTCGTTGGCA 3’; it spans coding exons 1 through 6. The probe was end-labeled with ^32^P-dCTP using Ready-To-Go DNA Labeling Beads (-dCTP) from GE Healthcare (formerly Amersham Biosciences, Piscataway, NJ). The labeled probe was column-purified using ProQuant G-50 Micro columns (GE Healthcare) and was hybridized to the Expression Array according to Clontech’s instructions. The most stringent final wash was in 0.1X SSC + 0.5% SDS at 66°C for 30 min. The array was exposed to X-ray film for 4 d. We determined the relative levels of WASH expression in different tissues by normalizing hybridization intensities on the dot blot to the hybridization intensities of a control ubiquitin probe as supplied by Clontech and labeled and hybridized to the dot-blot as described above. The ubiquitin-probed blot was washed with 0.1X SSC + 0.1% SDS at 55°C for 40 min, and exposed for 1 d.

Secondly, we hybridized a WASH probe to a Multiple Tissue Northern Blot (Clontech Inc., Mountain View CA). The probe was a 300-bp PCR product amplified from the WASH BC048328 cDNA clone using primers 5’ TAGAGTCCATCCGCCAAGC 3’ and 5’ GATTCCCAGTCGTCCTCGT 3’ situated in coding exons 8 and 10, respectively. The probe was labeled as described above and hybridized according to Clontech’s instructions, except that the blot was hybridized overnight. The most stringent wash was in 0.1x SSC + 0.1% SDS at 55°C for 40 min. The blot was exposed to X-ray film for 4 d. The blot was stripped and rehybridized with a 1.8-kb full-length β-actin cDNA control probe supplied by Clontech, labeled and hybridized as above, washed most stringently at 0.1X SSC + 0.1% SDS at 60°C for 45 min, and exposed for 1 d.

The hybridization intensities for both dot- and Northern blots were quantified using a Typhoon Phosphorimager (Amersham) and analyzed using ImageQuant software (Amersham). The ubiquitin-normalized dotblot results are consistent with publicly available microarray expression data using the probe gnf1h03908_s_at, which encompasses exon 9 of the human WASH gene, as a reporter^22^. Overall, hematopoietic tissues such as lymph node, thymus, and bone marrow as well as some brain areas appear to express higher levels of WASH than muscle and heart. The β-actin-normalized WASH gene expression measured using the Northern blot was highest in brain and peripheral blood leukocytes.

Thirdly, the expression results were confirmed by RT-PCR using RNA samples of human lymph node, liver, heart, and fetal brain supplied by Clontech. First-strand cDNA was reverse-transcribed from 2 μg of RNA by Superscript II (Invitrogen, Carlsbad, CA) using a gene-specific primer located in the 10th coding exon of WASH: 5’TGTCAGAGCAATGGCCCAA 3’. dNTPs were used at a final concentration of 2 mM. Dithiothreitol (DTT) (Invitrogen) was used at a final concentration of 8 mM as a reducing agent to minimize secondary structure of RNA as well as to retard any RNase activity. RNaseOUT (20 units, Invitrogen) was used as an RNase Inhibitor. The reverse transcription was performed at 37°C for 1 h in a thermocycler (PerkinElmer, Wellesley, MA) followed by reverse transcriptase inactivation at 95°C for 5 min. A 300-bp product was amplified by PCR from the resulting cDNA and confirmed by sequencing using the forward primer 5’ TAGAGTCCATCCGCCAAGC 3’ and reverse primer 5’GATTCCCAGTCGTCCTCGT 3’. PCR reactions contained 1x PCR buffer, 2 mM MgCl_2_, 0.2 mM each dNTP, 400 nM each primer, 2.5 units Biolase DNA polymerase, 1 µl DNA, with annealing temperature of 65°C and a 1-min 72°C extension and 35 cycles. A product of expected size and sequence was observed in all four tissues.

**Human WASH variants**

To characterize the protein coding potential of additional human paralogs of WASH, we evaluated WASH exons from the chromosomes in the NIGMS monochromosomal hybrid panel #2 (http://ccr.coriell.org/nigms/hybrids/hybrids.html) that contain WASH (all exons: 2,3,6,9,12,15,19,20; only coding exons 2-10: 16, X, Y) ^8^. Each cell line in this panel contains one copy of one human chromosome. We first performed PCR analyses of these chromosomes with primers specific for certain known mutations that disrupt or alter the full-length ORF (Table C). These primer pairs are expected to generate appropriately sized product only from WASH copies that contain the deleterious, not functional sequence at these variant sites. PCR conditions were as in previous paragraph, except that 30 ng hybrid cell DNA was used as template, and that the annealing temperature was 66°C for all primers, except the 406449 and chr2 pairs, for which 64°C was used. The presence of a PCR product was assayed on ethidium-bromide-stained 1% agarose gel. These analyses revealed that only chromosomes 9 and 20 in this hybrid panel might encode intact full-length WASH protein(s).

| Table C. Primers used to test for specific deleterious mutations | | | |
| --- | --- | --- | --- |
| Primer | Sequence | Expected product (~bp) | Positive chrom’s in hybrid panel |
| AK261(F) | GCCAAGTACCCTGCTCCAG | 335 | 15 |
| AK424(R)* | GGAAATTCCTTCAGCTTCTCC |  |  |
| BC459(F)* | GTAGCCGAGCCTCTCAAGA | 1900 | 2,3,6 |
| BC459(R) | GCTTGTTGAAGAGATCCGACA |  |  |
| 406449(F) | TTCCTGTGTGCGTGAGCA | 400 | 3,6,12,15,19 |
| 406449(R)* | GGTCCAGGAAGACATACTTCTTC |  |  |
| chr2(F)* | GCTGCTCTTCAACACCACCT | 700 | 2,12,19 |
| chr2788(R) | ATGTACATGAGGTCGTTGGCA |  |  |
| chr15(F) | GCCAAGTACCCTGCTCCAG | 350 | 3 |
| chr15(R)* | TCACGCACACAGGAAAGTCA |  |  |

*Asterisks mark the primers whose 3’ end distinguishes a known stop-causing SNP or in/del variant.

We sequenced coding exons 1-10 from the two hybrid cell lines containing chromosome 9 or 20. For chromosome 9, a genomic PCR product of ~3.7 kb was generated using primers exon2(F) and exon10(R) (Table D). PCR conditions were 1x Bioline PCR buffer, 2 mM MgCl_2_, 0.5 mM dNTPs, 400 nM each primer, and 2.5 units Biolase DNA polymerase and 95°C for 5 min; 30 cycles of 94°C for 30s, 65°C for 30s, and 72°C for 4 min; 10 min at 72°C; hold at 4°C. The sequence of the bulk PCR products from the hybrid cell lines revealed the presence of two gene copies on chromosome 20. Therefore, for chromosome 20, the two haplotypes spanning exons 2-10 were resolved by amplifying, TA-cloning and sequencing three exons at a time with one overlapping exon to connect the haplotypes. We generated PCR products spanning coding exons 2-4, 4-6, 6-8 and 8-10 using the primers in Table D. The PCR program for these amplifications was 5 min at 95°C; 35 cycles of 30 s at 94°C, 30 s at 65°C, 1.5 min at 72°C; 10 min at 72°C; hold at 4°C. We sequenced TA-cloned PCR products using the primers marked with ^S^ in Table D, BigDye v3 sequencing reagent and a 3730 DNA Analyzer (Applied Biosystems Inc., Foster City CA). We separately amplified, cloned and sequenced exon 1 from the chromosome 9 and 20 hybrids using exon1 primers (Table D).

| Table D. Primers used to PCR and sequence(^S^) WASH coding exons 2-10 | | |
| --- | --- | --- |
| Primer | Sequence | Expected product (bp) |
| exon1(F)^S^ | AGTTGGGATGAGGGCTGTG | 400 |
| exon1(R)^S^ | AAGTTGGGCTCTCACTTCAGC |  |
| exon2(F)^S^ | AGCCCAGCCTCTGTGATCTTC | 208 |
| exon2(R) | CTCAGAGGCACGGGGAAAT |  |
| exon4(F)^S^ | CAGCTGATTAGGCCCAGCAC | 264 |
| exon4(R) | GCCCTGGGTCCCCACTAA |  |
| exon6(F)^S^ | TGCAGGTCCCTGTCACCTC | 309 |
| exon6(R) | AGGGAAGAAGGTGTGTGAGCA |  |
| exon8(F)^S^ | CTGAGCAGCCTTTCCTCCTTG | 274 |
| exon8(R) | GGTGGACTCAGGGCTGGA |  |
| exon10(F) | CACCAACCACCCTCACTCAGC | 532 |
| exon10(R) | CAGTCACTGACCCCGAGACG |  |
| M13(F)^S^ | GTAAAACGACGGCCAG |  |
| M13(R)^S^ | CAGGAAACAGCTATGAC |  |

*Multi-exon products were generated using exon2(F)-exon4(R) pair, 880 bp; exon4(F)-exon6(R), 1020 bp; exon6(F)-exon8(R), 1380 bp; exon8(F)-exon10(R), 1660 bp.

Since PCR of a hybrid cell line provides no information about the chromosomal location of the analyzed sequence, we verified that WASH is located at 9pter and at both ends of chromosome 20 using FISH with the pool of probes A-E in the corresponding hybrid cell lines (GM10611 and GM13140).

To characterize interindividual variation in the WASH sequence coding potential of chromosomes 9 and 20, we amplified WASH from chromosomes separated by flow cytometry from lymphoblastoid cell lines of three unrelated human individuals (GM10494, GM07791, and GM10540 (see above)) and sequenced the PCR products. Chromosomes were prepared and sorted on an Influx sorter (Cytopeia, Inc., Seattle, WA) as described previously^23^. Chromosome 20 (both alleles) and a pool of chromosomes 9, 10, 11, and 12 (which are not resolvable by the combination of Hoechst 33258 and chromomycin A3 staining) were sorted from each individual into 25 µl UV-irradiated sterile H_2_O (5000 chromosomes per tube). The sorted chromosomes were subjected to several rounds of freezing and thawing (to improve DNA accessibility) and then amplified using the Genomiphi DNA Amplification Kit (Amersham Biosciences, Piscataway, NJ). The resulting amplified DNA was used to generate long-range PCR products encompassing coding exons 2-10 using forward primer 5’ TAGAGCAGAGCCGGAGCCAGGTGC 3’ and reverse primer 5’ GTCGTCCTCGTCCTCCTCTGCCTGTG 3’. PCR reactions included 1x HiFi buffer, 2 mM MgCl2, 0.5 mM each dNTP, 400 nM each primers, 1 µl DNA from the Genomiphi reaction, and 1 unit HiFi polymerase (Roche Diagnostics, Indianapolis, IN). The cycling program was the same as given above for long-range PCR reactions, except extension was performed at 68°C rather than 72°C. The resulting PCR products were cloned in the TOPO TA cloning vector (Invitrogen, Carlsbad, CA), and the clones were sequenced using BigDye v.3 with 3730 DNA Analyzer (Applied Biosystems Inc., Foster City, CA) with primers marked with ^S^ in Table D so as to obtain long-range haplotypes of sequence almost encompassing the nine exons. (Exon 1 was not included due to the difficulty of resolving haplotypes over the large genomic distance separating exons 1 and 2, Figure 1). We also generated long-range, exon 2-10 PCR products directly from genomic DNA of the three cell lines with HiFi polymerase as described above. The resulting PCR products were TA-cloned and sequenced as described above.

Due to the polymorphic distribution of WASH, the number of potentially different WASH copies in these sorted samples varies based on our FISH analyses of these individuals (chromosome 9-12 sort: 5, 6, and 6 in GM10494, GM07791, and GM10540 respectively; chromosome 20 sort: both ends of both homologs in all 3 individuals). The numbers of sequenced cloned PCR products, different haplotypes found, and different intact ORF sequences observed are in Table E. Supplementary Table 1 gives details of variant sites within the intact ORF sequences.

| Table E. WASH haplotype information | | | | |
| --- | --- | --- | --- | --- |
| Cell line | Template | number of sequenced clones | number of haplotypes | number of intact ORFs |
| GM10494 | genomic DNA | 10 | 6 | 3 |
|  | flow-sorted 20 | 9 | 2 | 1 |
|  | flow-sorted 9-12 | 6 | 5 | 1 |
| GM07791 | genomic DNA | 7 | 5 | 0 |
|  | flow-sorted 20 | 7 | 1 | 1 |
|  | flow-sorted 9-12 | 3 | 1 | 1 |
| GM10540 | genomic DNA | 15 | 10 | 2 |
|  | flow-sorted 20 | 3 | 1 | 1* |
|  | flow-sorted 9-12 | 4 | 2 | 1 |

*has same coding haplotype, but different intronic sequence as one of the intact haplotypes derived from genomic DNA template.

**REFERENCES CITED**

1.	Birney, E., Clamp, M. & Durbin, R. GeneWise and Genomewise. *Genome Res* 14, 988-995 (2004).

2.	Corpet, F. Multiple sequence alignment with hierarchical clustering. *Nucleic Acids Res* 16, 10881-10890 (1988).

3.	Schneider, T.D. & Stephens, R.M. Sequence logos: a new way to display consensus sequences. *Nucleic Acids Res* 18, 6097-6100 (1990).

4.	Crooks, G.E., Hon, G., Chandonia, J.M. & Brenner, S.E. WebLogo: a sequence logo generator. *Genome Res* 14, 1188-1190 (2004).

5.	Yang, Z. & Nielsen, R. Estimating synonymous and nonsynonymous substitution rates under realistic evolutionary models. *Mol Biol Evol* 17, 32-43 (2000).

6.	Yang, Z. PAML: a program package for phylogenetic analysis by maximum likelihood. *Comput Appl Biosci* 13, 555-556 (1997).

7.	Sawyer, S. Statistical tests for detecting gene conversion. *Mol Biol Evol* 6, 526-538. (1989).

8.	Linardopoulou, E.V. *et al.* Human subtelomeres are hot spots of interchromosomal recombination and segmental duplication. *Nature* 437, 94-100 (2005).

9.	Marchler-Bauer, A. & Bryant, S.H. CD-Search: protein domain annotations on the fly. *Nucleic Acids Res* 32, W327-331 (2004).

10.	Schultz, J., Milpetz, F., Bork, P. & Ponting, C.P. SMART, a simple modular architecture research tool: identification of signaling domains. *Proc Natl Acad Sci U S A* 95, 5857-5864 (1998).

11.	Letunic, I. *et al.* SMART 4.0: towards genomic data integration. *Nucleic Acids Res* 32, D142-144 (2004).

12.	Henikoff, S. & Henikoff, J.G. Protein family classification based on searching a database of blocks. *Genomics* 19, 97-107 (1994).

13.	Nakai, K. & Horton, P. PSORT: a program for detecting sorting signals in proteins and predicting their subcellular localization. *Trends in biochemical sciences* 24, 34-36 (1999).

14.	Tang, Z., El Far, O., Betz, H. & Scheschonka, A. Pias1 interaction and sumoylation of metabotropic glutamate receptor 8. *J Biol Chem* (2005).

15.	la Cour, T. *et al.* Analysis and prediction of leucine-rich nuclear export signals. *Protein Eng Des Sel* 17, 527-536 (2004).

16.	Miki, H., Suetsugu, S. & Takenawa, T. WAVE, a novel WASP-family protein involved in actin reorganization induced by Rac. *Embo J* 17, 6932-6941 (1998).

17.	Rosales-Nieves, A.E. *et al.* Coordination of microtubule and microfilament dynamics by Drosophila Rho1, Spire and Cappuccino. *Nat Cell Biol* 8, 367-376 (2006).

18.	Torok, T., Tick, G., Alvarado, M. & Kiss, I. P-lacW insertional mutagenesis on the second chromosome of Drosophila melanogaster: isolation of lethals with different overgrowth phenotypes. *Genetics* 135, 71-80 (1993).

19.	Trask, B.J. *et al.* Members of the olfactory receptor gene family are contained in large blocks of DNA duplicated polymorphically near the ends of human chromosomes. *Hum Mol Genet* 7, 13-26 (1998).

20.	Trask, B.J. *et al.* Large multi-chromosomal duplications encompass many members of the olfactory receptor gene family in the human genome. *Hum Mol Genet* 7, 2007-2020 (1998).

21.	Charchar, F.J. *et al.* Complex events in the evolution of the human pseudoautosomal region 2 (PAR2). *Genome Res* 13, 281-286 (2003).

22.	Su, A.I. *et al.* A gene atlas of the mouse and human protein-encoding transcriptomes. *Proc Natl Acad Sci U S A* 101, 6062-6067 (2004).

23.	Mefford, H.C., Linardopoulou, E., Coil, D., van den Engh, G. & Trask, B.J. Comparative sequencing of a multicopy subtelomeric region containing olfactory receptor genes reveals multiple interactions between non-homologous chromosomes. *Hum Mol Genet* 10, 2363-2372 (2001).
